# Fiber Rearrangement and Matrix Compression in Soft Tissues: Multiscale Hypoelasticity and Application to Tendon

**DOI:** 10.3389/fbioe.2021.725047

**Published:** 2021-10-12

**Authors:** Claire Morin, Christian Hellmich, Zeineb Nejim, Stéphane Avril

**Affiliations:** ^1^ Mines Saint-Etienne, Univ. Lyon, Univ. Jean Monnet, INSERM, U1059 Sainbiose, Centre CIS, Saint-Etienne, France; ^2^ Institute for Mechanics of Materials and Structures, TU Wien - Vienna University of Technology, Vienna, Austria

**Keywords:** multiscale hypoelasticity, micromechanics, homogenized stiffness, fiber decrimping, scale-dependent strain

## Abstract

It is widely accepted that the nonlinear macroscopic mechanical behavior of soft tissue is governed by fiber straightening and re-orientation. Here, we provide a quantitative assessment of this phenomenon, by means of a continuum micromechanics approach. Given the negligibly small bending stiffness of crimped fibers, the latter are represented through a number of hypoelastic straight fiber phases with different orientations, being embedded into a hypoelastic matrix phase. The corresponding representative volume element (RVE) hosting these phases is subjected to “macroscopic” strain rates, which are downscaled to fiber and matrix strain rates on the one hand, and to fiber spins on the other hand. This gives quantitative access to the fiber decrimping (or straightening) phenomenon under non-affine conditions, i.e. in the case where the fiber orientations cannot be simply linked to the macroscopic strain state. In the case of tendinous tissue, such an RVE relates to the fascicle material with 50 μm characteristic length, made up of crimped collagen bundles and a gel-type matrix in-between. The fascicles themselves act as parallel fibers in a similar matrix at the scale of a tissue-related RVE with 500 μm characteristic length. As evidenced by a sensitivity analysis and confirmed by various mechanical tests, it is the initial crimping angle which drives both the degree of straightening and the shape of the macroscopic stress-strain curve, while the final linear portion of this curve depends almost exclusively on the collagen bundle elasticity. Our model also reveals the mechanical cooperation of the tissue’s key microstructural components: while the fibers carry tensile forces, the matrices undergo hydrostatic pressure.

## 1 Introduction

With the advent of mechanobiology (Van der Meulen and Huiskes, 2002), it has been widely accepted that the behavior of cells and tissues is not only governed by genetic and chemical, but also by mechanical stimuli, such as mechanical stress (“force per area” typically expressed through Cauchy’s stress tensor) or mechanical strain (“length and angle changes,” mathematically expressed by any strain measure of the Seth-Hill family (Seth, 1962, 1966; Hill, 1968; Farahani and Naghdabadi, 2000), including the Green-Lagrange strain tensor representing “engineering strain” and the logarithmic strain tensor representing “true strain”). However, observing the mechanical stimuli may turn out as tricky, as they strongly depend on the length scale on which they are defined, i.e. on the size of the relevant areas and lengths. Hence, it is advisable to quantify the mechanical environment directly felt by the biological cells. By example, the oscillating hydrostatic pore pressure in bone stimulates osteoblasts and osteocytes (Klein-Nulend et al., 1995). These cell types, in turn, regulate tissue metabolism, i.e. the apposition of new bone tissue, or the resorption of old bone tissue, thereby changing the tissue morphology, in particular so the vascular porosity (Pastrama et al., 2018). This is the classical epitome of a mechanobiological process: mechanics-driven tissue regulation.

At the same time, there exists a more direct and even more profound type of mechanics-driven changes in tissue morphology, not even involving explicit cellular activities: the fiber reorientation and recruitment processes occurring in soft tissues (Lake et al., 2009; Gusachenko et al., 2012), with the aforementioned fibers being embedded in a gel-type substance (Weiss and Gardiner, 2001). These processes, in general, cannot be traced back to macroscopic deformations measured at the tissue scale (i.e. that of hundreds of micrometers in the case of tendons or arteries), but they are inherently linked to the mechanical environment of the fibers themselves, and of the soft gel-type matrix in-between theses fibers. Still, the fiber re-orientation and recruitment processes do not involve any explicit cellular activity, but merely the reaction of the hierarchically organized microstructure to mechanical forces. However, this reaction is a truly complex one, having challenged biomaterial mechanicians for decades now. In this context, a major challenge lies in the proper choice of a suitable deformation measure itself. The widely used Green-Lagrange strain tensor links any energetic state of the material microstructure to its initial configuration (Holzapfel et al., 2000), and corresponding material behaviors are often classified as “affine” (Gasser et al., 2006; Li et al., 2018), with interesting ranges of applicability (Holzapfel et al., 2002, Holzapfel et al., 2005; Kiousis et al., 2009; Pierce et al., 2010). Still, various experimental data on stress-strain behavior of soft tissues cannot be represented as explicit functions of the Green-Lagrange strain tensor (Criscione et al., 2003a; Criscione et al., 2003b) - this highlights the limitations of macroscopic hyperelasticity. As a remedy, Freed and coworkers (Freed, 2008, Freed, 2009, Freed, 2010; Freed et al., 2010; Freed and Einstein, 2012) proposed the use of macroscopic hypoelasticity for soft tissues: then objective, i.e. observer-independent, *rates* of macroscopic stress and strain tensors are linked to each other. The hypoelasticity concept was introduced by Truesdell (1955), and triggered intensive discussions (Bernstein, 1960b; Bernstein, 1960a; Xiao et al., 1997) on the integrability of relationships between stress and strain rates into either Cauchy-elasticity (where the Cauchy stress is a function of the deformation gradient) or Green-elasticity (also called hyperelasticity - where strain energy function depends on the Green-Lagrange strain tensor). As a rule, both Cauchy-elasticity and Green-elasticity turned out as special cases of hypoelasticity (Noll, 1955; Xiao et al., 1999), so that the physical nature of the latter remained somewhat open at that point in time. A major step forward was taken by Rajagopal and co-workers since 2003, by resorting to the thermodynamic definition of elasticity, i.e. to mechanical stress-driven, dissipation-free deformations (Rajagopal, 2003, Rajagopal, 2007; Rajagopal and Srinivasa, 2007, Rajagopal and Srinivasa, 2009; Rajagopal, 2011). They identified a class of non-dissipative, non-hyperelastic material models - with the hypoelastic models just being a subclass of those. As it was already the case in (Morin et al., 2018), this thermodynamic perspective on hypoelasticity is a major theoretical ingredient of the present paper. While being assured of the absence of any type of dissipation, this modeling approach does without the deformation gradient or the Green-Lagrange strain tensor. Accordingly, the current material behavior exclusively depends on the “here and now”, without any reference to the initial configuration.

Driving this philosophy to the next level of refinement, Morin et al. (2018) introduced hypoelasticity already at the microstructural level, thereby adopting an objective, thermodynamically consistent formulation based on the Gibbs potential (Rajagopal and Srinivasa, 2009, Rajagopal and Srinivasa, 2011): Strain rate and stress average rules (Hashin, 1983; Zaoui, 2002) arising from kinematic compatibility and mechanical equilibrium of material volumes representing soft tissue microstructures, together with Eshelby’s matrix-inclusion problem reformulated for velocity gradients (Morin et al., 2018), allowed for translating fiber deformations and re-orientations into macroscopically non-affine material behavior, in line with experimental observations (Goulam Houssen et al., 2011; Screen et al., 2004b; Gupta et al., 2010; Jayyosi et al., 2017; Krasny et al., 2017, Krasny et al., 2018). The present contribution tackles the next logical step: elucidating the nature of the associated macroscopic stiffness linking macroscopic Eulerian strain rates and objective stress rates; and hence, allowing for the establishment of hierarchical multiscale models, where the “macroscopic” stiffness properties arising from the homogenization over one (smaller) representative volume element (RVE) enter as (microstructural) phase properties within yet another (larger) RVE. At the same time, the micromechanical formulation allows for downscaling the strains subjected to an RVE, not only to fiber strains and re-orientations, but also to matrix strains. This allows for the detection of “unusual” material behavior, such as matrix compression under an overall uniaxial tensile stress state applied to the RVE. Accordingly, the paper is organized as follows: First, a continuum micromechanics framework for evolving elastic microstructures under large strains is established, with the following key ingredients: a representative volume element (RVE) obeying the scale separation principle and being subjected to homogeneous strain rate boundary conditions, thermodynamically consistent hypoelastic constitutive laws at the phase level; and matrix-inhomogeneity problems used for hypoelasticity upscaling (*see* Section 2). The following steps are then taken by example of tendinous tissue: After describing an algorithm for a hierarchical two-step homogenization scheme (*see* Section 3), micromechanical model results are presented in terms of sensitivity analyses and predictions of experimentally observed stress-strain relations, together with corresponding fiber re-orientations, fiber stretches, matrix stresses, and overall transverse stretches (*see* Section 4). The paper is concluded by a Discussion (*see* Section 5).

## 2 Continuum Micromechanics of Evolving Elastic Microstructures Undergoing Large Strains

### 2.1 Kinematics and Equilibrium

Continuum micromechanics provides estimates for the “homogenized” constitutive behavior of materials, from geometrical and mechanical information associated to their microstructures. Accordingly, these materials are considered to be, at the same time, micro-heterogeneous and macro-homogeneous. In this context, the material is seen as the matter filling a so-called representative volume element (RVE) of volume Ω, which satisfies the separation of scales principle, reading as (Hill, 1963; Drugan and Willis, 1996; Zaoui, 1997, Zaoui, 2002):

d≪ℓ≪L (1)

whereby *d*, *ℓ*, and L are respectively the characteristic lengths of the (micro-)heterogeneities, of the RVE, and of the structure built up by this material or of the loading applied to this structure. The latter “structural length” may be quantified through the spatial fluctuations of the macroscopic stresses **Σ** assigned to the macroscopic material points making up the structure, according to (Auriault et al., 2009);

$$L=\frac{\left\|\Sigma \right\|}{\left\|\partial \Sigma /\partial \underline{X}\right\|}$$ (2)

with $\underline{X}$ as the position vector labeling macroscopic material points within the given structure, e.g. within the considered organ.

**FIGURE 1:**
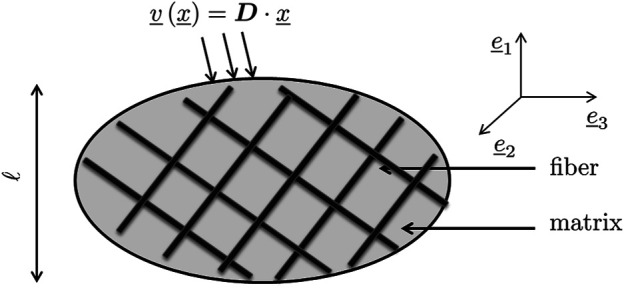
Representative volume element with characteristic length *ℓ*, subjected to homogeneous boundary conditions in terms of a microscopic velocity field arising from one macroscopic strain rate.

**FIGURE 2:**
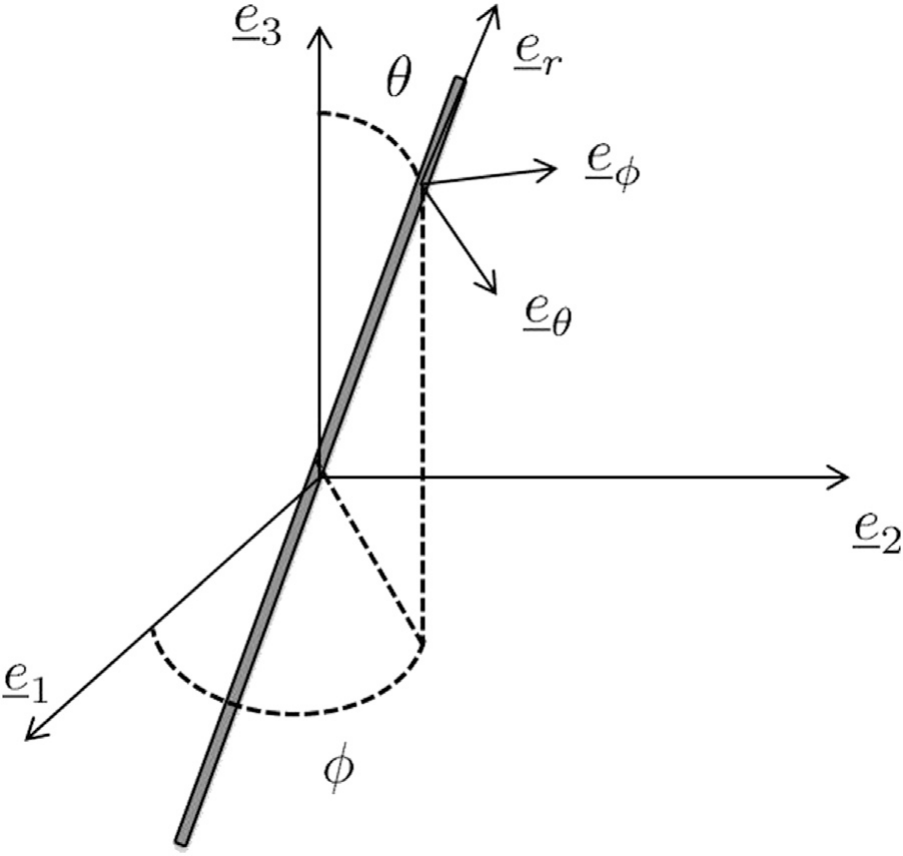
Definition of the local reference system ${\underline{e}}_{r},{\underline{e}}_{\theta },{\underline{e}}_{\phi }$ associated with a specific fiber phase; and associated Euler angles *θ* and *ϕ*.

Next, we adopt a statistical description of the microstructural morphology found within the RVE, in terms of homogeneous subdomains with given shape, volume fraction, and mechanical properties. These subdomains are called the material phases and provide an approximate description of the RVE. For the present case, illustrated in Figure 1, we consider *N*_*f*_ cylindrical phases with a length-to-diameter ratio going to infinity. These phases represent fibers (with volume fraction *f*_*r*_, *r* = 1, …, *N*_*f*_), and they are embedded into a soft matrix phase, with volume fraction $f_{m}=1-\sum_{r=1}^{N_{f}}f_{r}$ . The fiber orientations are quantified in terms of two Euler angles *θ* and *ϕ*, which define a local spherical coordinate system attached to the cylinder, as seen in Figure 2.

On the surface *∂*Ω of the RVE, the macroscopic strain rate is prescribed in terms of a microscopic velocity field $\underline{v}$ , reading mathematically as (Hashin, 1983; Morin et al., 2018):

$$\forall \underline{x}\in \partial \Omega :\;\underline{v}\left(\underline{x}\right)=D\cdot \underline{x}$$ (3)

with $\underline{x}$ as the microscopic location vector, *∂*Ω as the external boundary of the RVE with volume Ω, $\underline{v}$ as the prescribed velocity field, and ***D*** as the (Eulerian) macroscopic strain rate associated to macroscopic point $\underline{X}$ - a dependency which we will not explicitly indicate in the following developments, in order to keep the notation relatively compact. At the same time, we emphasize that ***D*** is independent of the microscopic location vector $\underline{x}$ (hence, Eq. 3 is referred to as “homogeneous boundary conditions”). We let the microscopic velocity field prescribed at the RVE’s boundary induce a continuous and differentiable velocity field $\underline{v}$ inside the RVE; and we express the corresponding velocity gradient in terms of strain rate and spin tensor fields, in accordance with their customary definitions as the symmetric and the skew-symmetric parts of the velocity gradient (Salençon, 2001):

$$\forall \underline{x}\in \Omega :\;d\left(\underline{x}\right)=\frac{1}{2}\left(\frac{\partial \underline{v}}{\partial \underline{x}}\left(\underline{x}\right)+{\left[\frac{\partial \underline{v}}{\partial \underline{x}}\right]}^{T}\left(\underline{x}\right)\right)$$ (4)

$$\forall \underline{x}\in \Omega :\;\omega \left(\underline{x}\right)=\frac{1}{2}\left(\frac{\partial \underline{v}}{\partial \underline{x}}\left(\underline{x}\right)-{\left[\frac{\partial \underline{v}}{\partial \underline{x}}\right]}^{T}\left(\underline{x}\right)\right)$$ (5)

The local spin and strain rates give access to the evolution of arbitrarily chosen local base vectors $\underline{e}$ attached to microscopic material points, *via* (Salençon, 2001):

$$\underline{\dot{e}}\left(\underline{x}\right)=\left[d\left(\underline{x}\right)+\omega \left(\underline{x}\right)\right]\cdot \underline{e}\left(\underline{x}\right)$$ (6)

with the dot operator referring to the time derivative.

The microscopic definitions of the Eulerian strain rate and spin, Eqs 4, 5, together with the homogeneous strain rate boundary condition Eq. 3, entail the following average rules (Hashin, 1983; Morin et al., 2018):

$$\frac{1}{\Omega }\int_{\Omega }d\left(\underline{x}\right)\;d\Omega =\left\langle d\right\rangle =\overset{N_{f}}{\underset{r=1}{\sum }}f_{r}d_{r}+f_{m}d_{m}=D,$$ (7)

$$\frac{1}{\Omega }\int_{\Omega }\omega \left(\underline{x}\right)\;d\Omega =\left\langle \omega \right\rangle =\overset{N_{f}}{\underset{r=1}{\sum }}f_{r}\omega_{r}+f_{m}\omega_{m}=0,$$ (8)

whereby the angular bracket notation ⟨⋅⟩ denotes the spatial average over the volume of the RVE; ***d***_*r*_ and ***ω***_*r*_ are the averages of ***d*** and ***ω*** over Ω_*r*_, the volume of the *r*-th fiber phase, while ***d***_*m*_ and ***ω***_*m*_ are the averages of ***d*** and ***ω*** over Ω_*m*_, the volume of the matrix phase. Moreover, the microscopic strain rates are considered to generate microscopic traction forces $\underline{t}$ at the boundary of the RVE and microscopic Cauchy stresses ***σ*** within the RVE. All these force quantities are equilibrated, which mathematically reads as:

$$\begin{array}{cc} & \forall \underline{x}\in \Omega :\;div\;\sigma \left(\underline{x}\right)=0\\ & \forall \underline{x}\in \partial \Omega :\;\underline{t}\left(\underline{x}\right)=\sigma \left(\underline{x}\right)\cdot \underline{n}\left(\underline{x}\right) \end{array}$$ (9)

with div as the divergence operator and *n* as the outward normal to the boundary at location $\underline{x}$ . The volume-normalized power of the external (traction) forces on the RVE, referred to in the sequel as external power density $p^{ext}$ , reads as (Morin et al., 2017):

$$\begin{array}{cc} p^{ext} & =\frac{1}{\Omega }\int_{\partial \Omega }\underline{t}\left(\underline{x}\right)\cdot \underline{v}\left(\underline{x}\right)\;dS\\ & =\frac{1}{\Omega }\int_{\partial \Omega }\left(D\cdot \underline{x}\right)\cdot \left[\sigma \left(\underline{x}\right)\cdot \underline{n}\left(\underline{x}\right)\right]dS\\ & =D:\frac{1}{\Omega }\int_{\Omega }\sigma \left(\underline{x}\right)\;d\Omega \end{array}$$ (10)

where use of the strain rate boundary condition Eq. 3 and of the equilibrium conditions Eq. 9 was made. Equation 10 induces a force quantity performing power (density) on the macroscopic strain rate ***D***, namely the macroscopic Cauchy stress **Σ**:

$$p^{ext}=\Sigma :D\Leftrightarrow \;\Sigma =\frac{1}{\Omega }\int_{\Omega }\sigma \left(\underline{x}\right)\;d\Omega =\overset{N_{f}}{\underset{r=1}{\sum }}f_{r}\sigma_{r}+f_{m}\sigma_{m}=\left\langle \sigma \right\rangle ,$$ (11)

with ***σ***_*r*_ and ***σ***_*m*_ as the averages of ***σ*** over Ω_*r*_ and Ω_*m*_, respectively. For the forthcoming developments, it is useful to consider all stress tensors appearing in Eq. 11 as being expressed in terms of components with respect to a fixed base frame ${\underline{e}}_{1},{\underline{e}}_{2},{\underline{e}}_{3}$ , *see* Figure 1, with indices written as superscripts,

$$\begin{array}{cc} \forall r\in \left\{1,‥,N_{f},m\right\}:\; & \sigma_{r}=\overset{3}{\underset{i=1}{\sum }}\overset{3}{\underset{j=1}{\sum }}\sigma_{r}^{ij}\;{\underline{e}}_{i}⊗{\underline{e}}_{j}\\ & \Sigma =\overset{3}{\underset{i=1}{\sum }}\overset{3}{\underset{j=1}{\sum }}\Sigma^{ij}\;{\underline{e}}_{i}⊗{\underline{e}}_{j} \end{array}$$ (12)

and then derive these components with respect to time, yielding a stress rate component average law of the format

$$\forall \left\{i,j\right\}\in \{1,2,3{\}}^{2}:\;\overset{N_{f}}{\underset{r=1}{\sum }}f_{r}\;{\dot{\sigma }}_{r}^{ij}+f_{m}\;{\dot{\sigma }}_{m}^{ij}={\dot{\Sigma }}^{ij}$$ (13)

### 2.2 Hypoelasticity

The matrix phase and the *N*_*f*_ different fiber phases making up the RVE exhibit a hypoelastic constitutive behavior (Truesdell, 1955). In more detail, the microscopic strain rate tensor ***d*** is linked to an objective rate of the microscopic stress tensor ***σ*** ^△^. For the sake of simplicity (Morin et al., 2018), we here choose the Jaumann rate, reading mathematically as (Jaumann, 1911; Szabó and Balla, 1989)

$$\sigma^{△}=\dot{\sigma }+\sigma \cdot \omega -\omega \cdot \sigma$$ (14)

since alternative, yet mathematically more laborious objective rates deliver, as a rule, very similar results (Morin et al., 2018). The link between strain and stress rate follows from the requirement of zero dissipation for elastic processes, which, when written as function of the Gibbs free energy per unit mass, $G_{\rho }\left(\sigma \right)$ , reads as (Rajagopal and Srinivasa, 2009, Rajagopal and Srinivasa, 2011; Morin et al., 2018):

$$D=\sigma :d-\sigma :\left(\rho \frac{\partial^{2}G_{\rho }}{\partial \sigma \partial \sigma }\right):\sigma^{△}=0$$ (15)

implying the following expression for the strain rate;

$$d=\rho \frac{\partial^{2}G_{\rho }}{\partial \sigma \partial \sigma }:\sigma^{△}$$ (16)

Equation 16 can be recast into the form originally given by Truesdell (1955):

$$\sigma^{△}=C:d$$ (17)

with the microscopic hypoelasticity (or stiffness) tensor being derived from the microscopic Gibbs free energy as:

$$C={\left(\rho \frac{\partial^{2}G_{\rho }}{\partial \sigma \partial \sigma }\right)}^{-1}$$ (18)

Considering homogeneous stiffness properties across the *N*_*f*_ fiber phases and the matrix phase,

$$\begin{array}{cc} \forall r\in \left\{1,‥,N_{f}\right\}:\;C_{r} & =\langle {C\rangle }_{\Omega_{r}}\\ C_{m} & =\langle {C\rangle }_{\Omega_{m}} \end{array}$$ (19)

we arrive at the following hypoelastic phase behavior:

$$\begin{array}{cc} \forall r\in \left\{1,‥,N_{f}\right\}:\;\sigma_{r}^{△} & =C_{r}:d_{r}\\ {\dot{\sigma }}_{m} & =C_{m}:d_{m} \end{array}$$ (20)

whereby we anticipated the vanishing spin of the matrix phase, ***ω***_*m*_ = 0, which, when specifying Eq. (14) for the matrix phase, yields:

$$\sigma_{m}^{△}={\dot{\sigma }}_{m}$$ (21)

### 2.3 Upscaling Hypoelasticity

The question arises of how to upscale the microscopic hypoelastic law Eq. 17 to the macroscopic level, i.e. to a relation linking macroscopic stress and strain measures. As a first step in this direction, we extend the reasoning of Zaoui (2002), by observing the linearity of the differential Equations 9, 17, which, together with boundary condition Eq. 3, imply a multi-linear relation between the macroscopic and microscopic strain rates:

$$\begin{array}{cc} \forall r\in \left\{1,‥,N_{f}\right\}:\;d_{r} & =A_{r}:D\\ d_{m} & =A_{m}:D \end{array}$$ (22)

with $A_{r}$ and $A_{m}$ as the fourth-order strain rate concentration tensors associated with the *r*-th fiber phase and with the matrix phase, respectively. The aforementioned linearity implies the existence of a similar relation for the spin, reading as:

$$\forall r\in \left\{1,‥,N_{f}\right\}:\;\omega_{r}=R_{r}:D$$ (23)

with $R_{r}$ as the fourth-order strain rate-to-spin concentration tensor associated with the *r*-th fiber phase.

Following again the tradition of continuum micromechanics (Zaoui, 2002), the mechanical interactions within the RVE depicted in Figure 1 are estimated by coupling a number of matrix-inhomogeneity problems in the sense of the famous paper of Eshelby (1957). Accordingly, the strain rate and strain rate-to-spin concentration tensors are estimated by means of a Mori-Tanaka scheme (Mori and Tanaka, 1973; Benveniste, 1987), following a strategy given in greater detail in (Morin et al., 2018):

$$\begin{array}{cc} \forall r\in \left\{1,‥,N_{f}\right\}:\;A_{r} & =A_{r}^{\infty }:{\left[\overset{N_{f}}{\underset{i=1}{\sum }}f_{i}A_{i}^{\infty }+f_{m}A_{m}^{\infty }\right]}^{-1}\\ \forall r\in \left\{1,‥,N_{f}\right\}:\;R_{r} & =R_{r}^{\infty }:{\left[\overset{N_{f}}{\underset{j=1}{\sum }}f_{j}A_{j}^{\infty }+f_{m}A_{m}^{\infty }\right]}^{-1}\\ with\forall r\in \left\{1,‥,N_{f}\right\}:\;A_{r}^{\infty } & ={\left[I+P_{r}:\left(C_{r}-C_{m}\right)\right]}^{-1}\\ \forall r\in \left\{1,‥,N_{f}\right\}:\;R_{r}^{\infty } & =-R_{r}^{Esh}:C_{m}^{-1}:{\left[I+\left(C_{r}-C_{m}\right):P_{r}\right]}^{-1}:\left(C_{r}-C_{m}\right) \end{array}$$ (24)

In Eq. 24, the following physical quantities are introduced: I is the fourth-order unity tensor, $P_{r}=S_{r}^{Esh}:C_{m}^{-1}$ is the Hill tensor of the *r*-th fiber phase. $S_{r}^{Esh}$ is the classical Eshelby tensor: within an infinite 3D domain exhibiting the elastic properties of the matrix, this fourth-order tensor relates an eigenstrain rate acting on an inclusion representing the *r*-th fiber phase, with the corresponding total strain rates in that inclusion. $R_{r}^{Esh}$ is an Eshelby-like tensor extending Eshelby’s original ideas towards spins: it relates an eigenstrain rate acting on an inclusion representing the *r*-th fiber phase with the corresponding spin of that inclusion. In a base frame ${\underline{e}}_{r},{\underline{e}}_{\theta },{\underline{e}}_{\phi }$ , being aligned with the direction of the *r*-th fiber phase, *see* Figure 2, the non-zero components of the aforementioned tensors read as (Eshelby, 1957; Morin et al., 2018):

$$\begin{array}{ccc} & & S_{\theta \theta \theta \theta }^{Esh}=S_{\phi \phi \phi \phi }^{Esh}=\frac{5-4\nu_{m}}{8\left(1-\nu_{m}\right)}\\ & & S_{\theta \theta \phi \phi }^{Esh}=S_{\phi \phi \theta \theta }^{Esh}=\frac{-1+4\nu_{m}}{8\left(1-\nu_{m}\right)}\\ & & S_{\theta \theta rr}^{Esh}=S_{\phi \phi rr}^{Esh}=\frac{\nu_{m}}{2\left(1-\nu_{m}\right)}\\ & & S_{\phi r\phi r}^{Esh}=S_{r\phi r\phi }^{Esh}=S_{r\phi \phi r}^{Esh}=S_{\phi rr\phi }^{Esh}=S_{r\theta r\theta }^{Esh}=S_{\theta r\theta r}^{Esh}=S_{\theta rr\theta }^{Esh}=S_{r\theta \theta r}^{Esh}=\frac{1}{4}\\ & & S_{\theta \phi \theta \phi }^{Esh}=S_{\phi \theta \phi \theta }^{Esh}=S_{\phi \theta \theta \phi }^{Esh}=S_{\theta \phi \phi \theta }^{Esh}=\frac{3-4\nu_{m}}{8\left(1-\nu_{m}\right)} \end{array}$$ (25)

$$\begin{array}{ccc} & & R_{\theta rr\theta }^{Esh}=R_{\phi rr\phi }^{Esh}=R_{\theta r\theta r}^{Esh}=R_{\phi r\phi r}^{Esh}=-\frac{1}{4}\\ & & R_{r\theta r\theta }^{Esh}=R_{r\phi r\phi }^{Esh}=R_{r\theta \theta r}^{Esh}=R_{r\phi \phi r}^{Esh}=\frac{1}{4} \end{array}$$ (26)

where $\nu_{m}=-{\left(C_{m}^{-1}\right)}^{\theta \theta \phi \phi }/{\left(C_{m}^{-1}\right)}^{\theta \theta \theta \theta }$ refers to the elastic Poisson’s ratio of the isotropic matrix into which the fiber phase oriented in direction ${\underline{e}}_{r}$ is embedded. The strain concentration tensor of the matrix phase, $A_{m}$ , follows from evaluation of Eq. 24_1_ and Eq. 24_3_ for *r* = *m*, yielding in particular $A_{m}^{\infty }=I$ . It is also helpful to evaluate Eq. 24_4_ for *r* = *m*, yielding $R_{m}^{\infty }=R_{m}=0$ , a result which we have already anticipated in Eqs 20, 21.

Inserting the two concentration relations Eq. 21 and Eq. 23, as well as the Jaumann rate Eq. 14, into the constitutive relation Eq. 17, yields a relation which links the microscopic phase-specific stress tensor components with respect to a fixed base at the current time instant, to both the macroscopic strain rate tensor *and* the microscopic stresses themselves. This reads mathematically as:

$$\begin{array}{cc} \forall r\in \left\{1,‥,N_{f}\right\}:\;{\dot{\sigma }}_{r} & =C_{r}:A_{r}:D-\sigma_{r}\cdot \left(R_{r}:D\right)+\left(R_{r}:D\right)\cdot \sigma_{r}\\ {\dot{\sigma }}_{m} & =C_{m}:A_{m}:D \end{array}$$ (27)

It is useful and illustrative to recast the expression Eq. 27 in index notation (with the indices being written as superscripts):

$$\begin{array}{cc} & \forall \left\{i,j\right\}\in {\left\{1,2,3\right\}}^{2}:\\ \forall r\in \left\{1,‥,N_{f}\right\}:\;{\dot{\sigma }}_{r}^{ij}= & \left[c_{r}^{ijkl}A_{r}^{lkmn}-\sigma_{r}^{ik}R_{r}^{kjmn}+R_{r}^{ikmn}\sigma_{r}^{kj}\right]D^{nm}\\ {\dot{\sigma }}_{m}^{ij}= & c_{m}^{ijkl}A_{m}^{lkmn}D^{nm} \end{array}$$ (28)

whereby the Einstein convention on repeated indices is adopted. Insertion of this expression into the stress component rate average law Eq. 13 yields an expression linking macroscopic stress rates to macroscopic strain rates, reading as:

$$\begin{array}{cc} & \forall \left\{i,j\right\}\in {\left\{1,2,3\right\}}^{2}:\;\\ {\dot{\Sigma }}^{ij} & =\left[\overset{N_{f}}{\underset{r=1}{\sum }}f_{r}\left(c_{r}^{ijkl}A_{r}^{lkmn}-\sigma_{r}^{ik}R_{r}^{kjmn}+R_{r}^{ikmn}\sigma_{r}^{kj}\right)+f_{m}c_{m}^{ijkl}A_{m}^{lkmn}\right]D^{nm} \end{array}$$ (29)

which induces a homogenized stiffness tensor with the following components (indices written as superscripts):

$$\begin{array}{cc} & \forall \left\{i,j,m,n\right\}\in {\left\{1,2,3\right\}}^{4}:\\ & C_{hom}^{ijmn}=\overset{N_{f}}{\underset{r=1}{\sum }}f_{r}\left(c_{r}^{ijkl}A_{r}^{lkmn}-\sigma_{r}^{ik}R_{r}^{kjmn}+R_{r}^{ikmn}\sigma_{r}^{kj}\right)+f_{m}c_{m}^{ijkl}A_{m}^{lkmn} \end{array}$$ (30)

This homogenized stiffness $C_{hom}$ exhibits several peculiar, particularly non-classical features: It shows only *minor* symmetry properties, i.e. $C_{hom}^{ijkl}=C_{hom}^{ijlk}=C_{hom}^{jikl}=C_{hom}^{jilk}$ , associated with the symmetry of the involved stress and strain tensors. Moreover, it depends not only on morphological features and microscopic stiffness properties, as quantified through the first term of the right-hand side of Eq. 30, but also on the microscopic stress states, in conjunction with the strain rate-to-spin concentration tensors $R_{r}$ . The latter are symmetric with respect to the two first indices and skew-symmetric with respect to the two last indices, i.e. $R_{r}^{ijkl}=R_{r}^{jikl}=-R_{r}^{ijlk}$ .

## 3 Hierarchically Organized Fibrous Microstructures in Tendinous Tissue

### 3.1 Sequence of RVEs and Phase Properties

**FIGURE 3:**
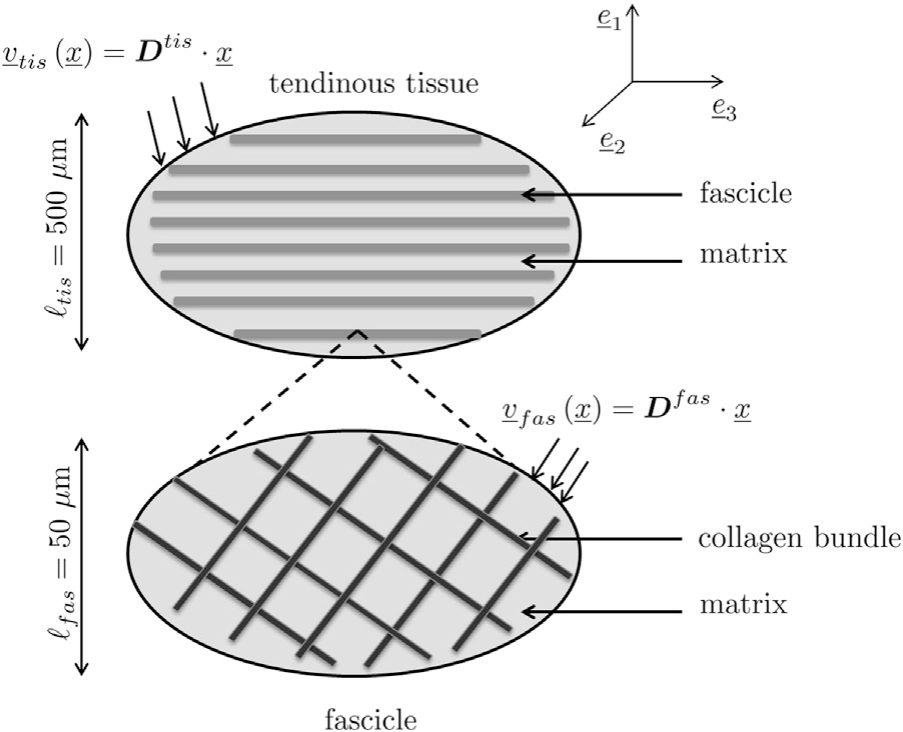
Two-scale micromechanical representation of tendinous tissue: **(top)** RVE of tendinous tissue, made up by fascicle phase embedded into gel-type matrix phase; **(bottom)** fascicle-related RVE made up by straight collagen bundle phases oriented in different directions and also embedded into a gel-type matrix phase.

The fibers introduced as phases within an RVE may not exhibit invariant material properties, but properties arising from yet another fibrous microstructure found within the aforementioned fiber phases. This is the case with tendinous tissue where parallel fibers called fascicles, with lengths spanning over several millimeters and 200 microns diameter (Niven et al., 1982; Kastelic et al., 1978), are made up by crimped collagen bundles, with lengths spanning over several millimeters and 100–300 nm diameter (Kastelic et al., 1978; Birk and Trelstad, 1986; Provenzano and Vanderby, 2006). Both types of fibers, the fascicles and the collagen bundles, are embedded into a gel-type matrix. This situation calls for the introduction of two types of RVEs at different scales, *see* Figure 3: An RVE with a characteristic size of *ℓ*_*tis*_ = 500 microns is associated with tendinous tissue (labelled by the subscript *tis*), and made up of parallel fibers making up a fascicle phase (labelled by the subscript *fas*) with a characteristic size of 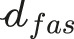
= 200 microns, being embedded into a matrix phase (labelled by the subscript *m*). The material making up the fascicle phase is represented by yet another RVE with a characteristic size *ℓ*_*fas*_. The latter needs to fulfill the size condition *ℓ*_*fas*_ ≤ 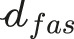
(Fritsch and Hellmich, 2007), as this RVE exhibits the homogeneous material properties of the fascicle phase. This fascicle-related RVE is made up of collagen bundles (labelled by the subscript *col*) with a characteristic size of 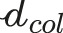
= 100…300 nm embedded in a soft matrix (labelled by the subscript *μ*). The collagen bundles are crimped (Abrahams, 1967; Kastelic et al., 1978; Hansen et al., 2002), and in order to represent this situation in the framework of the RVE seen in Figure 1, we introduce differently oriented straight fiber phases, all associated with mean initial crimping angle $\theta_{col}^{fas}\left(t=0\right)$ , with *t* = 0 indicating the start of the mechanical loading. The relevance of this modeling strategy arises from the very low bending stiffness of collagen bundles. In more detail, AFM-based micromechanical bending tests on single electron-spun or bovine Achilles tendon-derived collagen type I fibrils exhibit an apparent bending modulus of 0.1 …0.3 MPa (Yang et al., 2008b,a). Values of this magnitude are negligible with respect to the stretching stiffness of collagen type I bundles, amounting to 500 MPa according to X-ray-assisted tensile testing (Sasaki and Odajima, 1996a).

The larger RVE is subjected to tissue-related macroscopic strain rates ***D*** ^*tis*^, while the smaller RVE is subjected to fascicle-related macroscopic strain rates ***D*** ^*fas*^, which are identical to the fascicle phase-related strain rates $d_{fas}^{tis}$ ; hence, $D^{fas}\equiv d_{fas}^{tis}$ .

This hierarchical representation is complemented by the following phase properties (concerning elasticity, volume fractions, and fiber orientations):

• The bundle phase and both matrix phases exhibit a hypoelastic constitutive behavior according to Eq. 17. Moreover, for the sake of simplicity, they are considered to behave isotropically, so that:

C=3kJ+2μK (31)

with *k* and *μ* as the bulk and shear moduli, and J and K as the spherical and deviatoric parts of the fourth-order identity tensor respectively. The elastic isotropic constants *k* and *μ* can also be expressed in terms of the Young’s modulus *E* and of the Poisson’s ratio *ν*, through:

$$\begin{array}{cc} k & =\frac{E}{3\left(1-2\nu \right)}\\ \mu & =\frac{E}{2\left(1+\nu \right)} \end{array}$$ (32)

The collagen bundles exhibit a Young’s modulus of *E*_*col*_ = 500 MPa, according to the X-ray-assisted tensile tests of Sasaki and Odajima (1996a) on hydrated collagen fibrils of a bovine tendon; and a Poisson’s ratio of *ν*_*col*_ = 0.34, as obtained from acoustic experiments (Cusack and Miller, 1979; Vass et al., 2017; Morin et al., 2018). The two matrices are characterized by the same elastic constants, defined through a Young’s modulus of *E*_*m*_ = *E*_*μ*_ = 2.5 MPa, arising from a few micrometer deep nanoindentations in hyaline cartilage, a tissue with a large gel-type matrix volume fraction and non-recruited, disordered fibers (Franke et al., 2007). Motivated by the aforementioned acoustic tests as rare examples of Poisson’s ratio measurements on soft tissues at low length scales, we assign the value of *ν*_*m*_ = 0.34 also to the two matrix phases, depicted in Figure 3. As a further justification for this choice, we refer to Poisson’s ratio measurements on polymer gels and polyvinylalcohol gels, which indeed deliver similar experimental values (Li et al., 1993; Urayama et al., 1993).

• Image processing allows for the determination of the volume fraction of each phase: at the lower scale, processing transmission electron microscopy (TEM) images showing cross-sections of fascicles give access to the volume fraction of collagen bundles inside a fascicle, $f_{fas}^{tis}$ ; amounting to 0.95 according to Figure 3 of (Patterson-Kane et al., 2012). A collection of TEM results, as documented in Table 1, shows that the volume fraction of the bundles within a fascicle-related RVE, $f_{col}^{fas}$ , ranges between 0.6 and 0.9.

**TABLE 1:** Volume fractions of collagen bundles within a fascicle-related RVE, determined from transmission electron micrographs (TEM) of transverse cross sections taken across different species and anatomical locations.

Reference	Tendon	Species	Segmentation procedure	Volume fraction [−]
Screen et al. (2005)	tail	rats	tophat filter and contrast enhancement	0.83
Goh et al. (2008)	tail	mice (1.6)	None	0.56
Goh et al. (2008)	tail	mice (2.6)	None	0.79
Goh et al. (2008)	tail	mice (4)	None	0.85
Goh et al. (2008)	tail	mice (11.5)	None	0.78
Goh et al. (2008)	tail	mice (23)	None	0.76
Goh et al. (2008)	tail	mice (29)	None	0.81
Goh et al. (2008)	tail	mice (31.5)	None	0.78
Goh et al. (2008)	tail	mice (35.3)	None	0.76
Juneja and Veillette (2013)	tail	mice	tophat filter	0.80
Patterson-Kane et al. (2012)	SDFT	horse	median filter	0.72
Parent et al. (2011)	tail	adult rats	None	0.54
Pingel et al. (2014)	Achilles	human	median filter	0.62
Hansen et al. (2010)	ACL	human	tophat filter and contrast enhancement	0.68
Hansen et al. (2009)	PT	human	contrast enhancement and median filter	0.76

SDFT, superior digital flexor tendon; PT, patellar tendon; ACL, anterior cruciate ligament. Age of the mice in months is reported between brackets.

**FIGURE 4:**
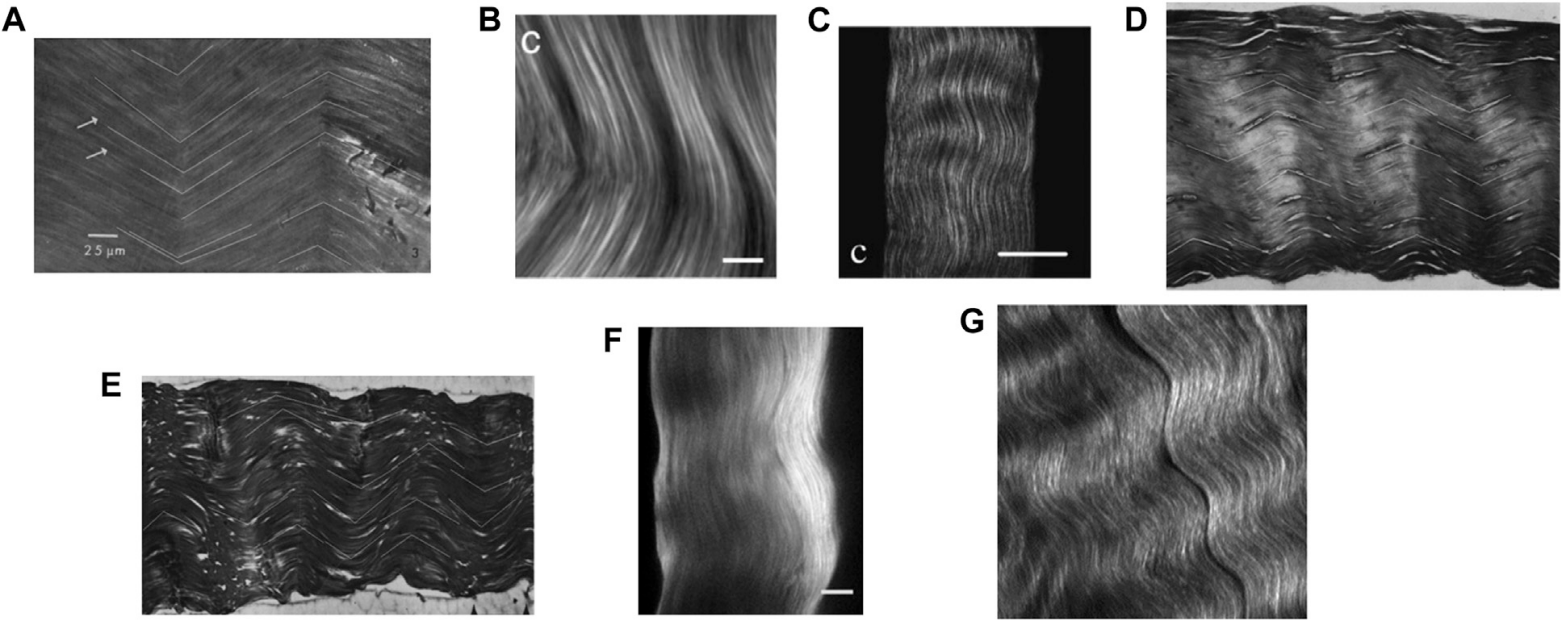
Microscopic images of tendinous tissue: **(A)** rat tail tendon in transmitted light, copied from the Figure 3 copied from (Dlugosz et al., 1978), copyright granted by Elsevier LTD, scale bar: 25 microns; **(B)** forward second harmonic generation (SHG) image of a mature Sprague-Dawley rat tail tendon, copied from the Figure 5C of (Williams et al., 2005), copyright granted by Elsevier LTD, scale bar: 10 microns; **(C)** SHG polarization from individual bundles analyzed with an analyzer oriented parallel to the rat tail tendon bundle, copied from the Figure 2C of (Williams et al., 2005), copyright granted by Elsevier LTD, scale bar: 50 microns; **(D**,**E)** histological longitudinal-sections of a rat tail tendon fascicle, copied from the Figures 4A,B of (Niven et al., 1982), copyright granted by Elsevier LTD, respective image width: 530 and 740 microns; **(F)** SHG imaging of the rat tail tendon fascicle microstructure after few cycles preconditioning, copied from Figure 2C of (Goulam Houssen et al., 2011), copyright granted by Elsevier LTD, scale bar: 50 microns; **(G)** porcine knee posterior cruciate ligament SHG image, copied from Figure 3 of (Lee et al., 2017), copyright granted by SPIE and agreement from the corresponding author, image width: 90 microns.

• Finally, image processing also gives access to the orientation of the fiber-type fascicle and bundle phases: Within an RVE of tendinous tissue, the fascicles are initially parallel and oriented in the axial direction, i.e. $\theta_{fas}^{tis}\left(t=0\right)=0$ , with time point *t* = 0 referring to a (still unloaded) situation at the beginning of the mechanical loading. In this case, the value of the longitudinal angle *ϕ* does not matter. Within the fascicle-related RVE, the angle $\theta_{col}^{fas}$ corresponds to the crimp angle, which can be measured *via* image processing as reported in Figure 4. Accordingly, this latitudinal angle $\theta_{col}^{fas}$ ranges between 15 and 45°. In this context, the longitudinal angle does matter. Since the fibers are crimped in the 3D space (De Campos Vidal, 2003; Kalson et al., 2012), four different values are introduced, $\phi_{col,r}^{fas}=0,90,180,270$ °; and they are associated with four collagen bundle phases, the behavior of which is fully identical.

### 3.2 Algorithm for Two-step Homogenization

The large deformation and the morphology evolution render the problem highly non linear and require the development of an incremental algorithm. Therefore the time line is discretized into time increments Δ*t*. These increments are bounded by time points *t*_*n*_, *n* = 1, ‥, *N*_*t*_, so that:

$$\Delta t=t_{n+1}-t_{n}\;forn=1,‥,N_{t}$$ (33)

The implicit dependence of the concentration operators on the orientation of the fiber phases asks for an explicit scheme for all time derivatives, which is defined as follows:

$$\dot{a}\left(t_{n}\right)=\frac{a\left(t_{n+1}\right)-a\left(t_{n}\right)}{\Delta t}$$ (34)

Assuming that the problem has been solved until time step *t*_*n*_ (with a known corresponding configuration), we have to determine the configuration at time step *t*_*n*+1_, as well as all associated mechanical and morphological quantities, and we proceed as follows:

1. We collect properties and operators known at time point *t*_*n*_: for the tendinous tissue-related RVE, we have $A_{fas}^{tis}\left(t_{n}\right),R_{fas}^{tis}\left(t_{n}\right)$ , and $A_{m}^{tis}\left(t_{n}\right)$ , they all depend on the stiffnesses $C_{fas}^{tis}\left(t_{n}\right)$ and $C_{m}^{tis}\left(t_{n}\right)$ as well as on the orientation $\theta_{fas}^{tis}\left(t_{n}\right)$ . However, in the present paper, we abstain from modeling fascicle re-orientation due to unusual load cases. Such reorientation modeling would require the introduction of multiple fascicle phases, which is beyond the scope of the present paper. On the other hand, for the fascicle-related RVE, we have collagen bundle phase-specific concentration tensors $A_{col,r}^{fas}\left(t_{n}\right)$ and $R_{col,r}^{fas}\left(t_{n}\right)$ , as well as the matrix-related concentration tensor $A_{\mu }^{fas}\left(t_{n}\right)$ ; all these tensors depend on the latitudinal angles $\theta_{col,r}^{fas}\left(t_{n}\right)$ .
2. This allows for estimating the phase-related strain rates and spins, by specifying Eq. 22 and Eq. 23 for the two-step homogenization scheme depicted in Figure 3:

$$\begin{array}{cc} d_{fas}^{tis}\left(t_{n}\right) & =A_{fas}^{tis}\left(t_{n}\right):D^{tis}\left(t_{n}\right)\\ d_{m}^{tis}\left(t_{n}\right) & =A_{m}^{tis}\left(t_{n}\right):D^{tis}\left(t_{n}\right) \end{array}$$ (35)

$$\begin{array}{cc} d_{col,r}^{fas}\left(t_{n}\right) & =A_{col,r}^{fas}\left(t_{n}\right):d_{fas}^{tis}\left(t_{n}\right)\\ \omega_{col,r}^{fas}\left(t_{n}\right) & =R_{col,r}^{fas}\left(t_{n}\right):d_{fas}^{tis}\left(t_{n}\right)\\ d_{\mu }^{fas}\left(t_{n}\right) & =A_{\mu }^{fas}\left(t_{n}\right):d_{fas}^{tis}\left(t_{n}\right) \end{array}$$ (36)

3. As a direct consequence, the orientation change of each collagen bundle phase within a fascicle-related RVE can be estimated based on discretized versions of Eq. 6, reading as:

$$\begin{array}{cc} & \forall \;{\underline{e}}_{j}\text{ attached to the }r-\text{th collagen bundle phase }\\ & {\underline{e}}_{j}\left(t_{n+1}\right)={\underline{e}}_{j}\left(t_{n}\right)+\left(d_{col,r}^{fas}\left(t_{n}\right)+\omega_{col,r}^{fas}\left(t_{n}\right)\right)\cdot {\underline{e}}_{j}\left(t_{n}\right)\;\Delta t \end{array}$$ (37)

4. The constitutive relation Eq. 20, in combination with Eq. 14, is discretized and specified for the two-step homogenization scheme of Figure 3, providing access to the updated stress state in the phases, at time *t*_*n*+1_. This reads for the tendinous tissue-related RVE as:

$$\begin{array}{cc} & \sigma_{fas}^{tis}\left(t_{n+1}\right)=\sigma_{fas}^{tis}\left(t_{n}\right)+\\ & \left(C_{fas}^{tis}\left(t_{n}\right):d_{fas}^{tis}\left(t_{n}\right)-\sigma_{fas}^{tis}\left(t_{n}\right)\cdot \omega_{fas}^{tis}\left(t_{n}\right)+\omega_{fas}^{tis}\left(t_{n}\right)\cdot \sigma_{fas}^{tis}\left(t_{n}\right)\right)\Delta t\\ & \sigma_{m}^{tis}\left(t_{n+1}\right)=\sigma_{m}^{tis}\left(t_{n}\right)+C_{m}^{tis}\left(t_{n}\right):d_{m}^{tis}\left(t_{n}\right)\Delta t \end{array}$$ (38)

and for the fascicle-related RVE as:

$$\begin{array}{cc} & \sigma_{col,r}^{fas}\left(t_{n+1}\right)=\sigma_{col,r}^{fas}\left(t_{n}\right)+\\ & \left(C_{col}^{fas}\left(t_{n}\right):d_{col,r}^{fas}\left(t_{n}\right)-\sigma_{col,r}^{fas}\left(t_{n}\right)\cdot \omega_{col,r}^{fas}\left(t_{n}\right)+\omega_{col,r}^{fas}\left(t_{n}\right)\cdot \sigma_{col,r}^{fas}\left(t_{n}\right)\right)\Delta t\\ & \sigma_{\mu }^{fas}\left(t_{n+1}\right)=\sigma_{\mu }^{fas}\left(t_{n}\right)+C_{\mu }^{fas}\left(t_{n}\right):d_{\mu }^{fas}\left(t_{n}\right)\Delta t \end{array}$$ (39)

In these equations, we identified the material derivative with the partial derivative, according to the first-order approximations detailed in (Morin et al., 2018).

5. In addition, the homogenized stiffness according to Eq. 30 is specified for both the fascicle-related and the tendinous tissue-related RVE, reading as:

$$\begin{array}{cc} C_{fas}^{ijmn}\left(t_{n+1}\right) & =f_{\mu }^{fas}C_{\mu }^{ijkl}\left(t_{n+1}\right)A_{\mu }^{fas,lkmn}\left(t_{n+1}\right)+\overset{N_{f}}{\underset{s=1}{\sum }}f_{col,s}^{fas}\left[C_{col,s}^{ijkl}\left(t_{n+1}\right)A_{col,s}^{fas,lkmn}\left(t_{n+1}\right)\right.\\ & \left.-\sigma_{col,s}^{fas,ik}\left(t_{n+1}\right)R_{col,s}^{fas,kjmn}\left(t_{n+1}\right)+R_{col,s}^{fas,ikmn}\left(t_{n+1}\right)\sigma_{col,s}^{fas,kj}\left(t_{n+1}\right)\right]\\ C_{tis}^{ijmn}\left(t_{n+1}\right) & =f_{m}^{tis}C_{m}^{ijkl}\left(t_{n+1}\right)A_{m}^{tis,lkmn}\left(t_{n+1}\right)+f_{fas}^{tis}\left[C_{fas}^{ijkl}\left(t_{n+1}\right)A_{fas}^{tis,lkmn}\left(t_{n+1}\right)\right.\\ & \left.-\sigma_{fas}^{tis,ik}\left(t_{n+1}\right)R_{fas}^{tis,kjmn}\left(t_{n+1}\right)+R_{fas}^{tis,ikmn}\left(t_{n+1}\right)\sigma_{fas}^{tis,kj}\left(t_{n+1}\right)\right] \end{array}$$ (40)

whereby $C_{fas}\equiv C_{fas}^{tis}$ .

6. Finally, the macroscopic stress is computed at time *t*_*n*+1_ according to:

$$\Sigma^{tis}\left(t_{n+1}\right)=\Sigma^{tis}\left(t_{n}\right)+C_{tis}\left(t_{n}\right):D^{tis}\left(t_{n}\right)\Delta t$$ (41)

In case the macroscopic stress **Σ** ^*tis*^, rather than the macroscopic strain rate ***D*** ^*tis*^, is prescribed, an estimate of the corresponding effective strain rate is computed as:

$$D^{tis,est}\left(t_{n}\right)=\frac{1}{\Delta t}{\left[C_{tis}\left(t_{n}\right)\right]}^{-1}:\left(\Sigma^{tis}\left(t_{n+1}\right)-\Sigma^{tis}\left(t_{n}\right)\right)$$ (42)

This estimate then enters the aforementioned algorithm, namely *via* Eq. 35, and the resulting stress according to Eq. 41 is compared to the applied stress. If the corresponding stress difference exceeds a prescribed error threshold, a new estimate for ***D*** ^*tis*^ is computed by means of a modified verison of Eq. 40, where the latest estimate for the tissue stiffness according to Eq. 40 is used. This process is repeated until the aforementioned stress difference becomes negligibly small.

It is illustrative to document corresponding model predictions in terms of stretches. The stretch associated with a line element which is originally oriented in direction ${\underline{e}}_{i}$ , is computed from the deformation gradient tensor, ***F***, as follows:

$$\lambda_{i}\left(t_{n},{\underline{e}}_{i}\right)={\left({\underline{e}}_{i}\cdot F^{T}\left(t_{n}\right)\cdot F\left(t_{n}\right)\cdot {\underline{e}}_{i}\right)}^{1/2}$$ (43)

whereby the deformation gradient tensor itself is computed from the strain rate and spin tensors (respectively ***D*** and **Ω**):

$$F\left(t_{n}\right)=\left(\left[D\left(t_{n-1}\right)+\Omega \left(t_{n-1}\right)\right]\Delta t+1\right)\cdot F\left(t_{n-1}\right)$$ (44)

These equations can be specialized for the cases of the axial and transverse stretches of tendinous tissue undergoing a uniaxial stress of the form $\Sigma^{tis}=\Sigma_{33}^{tis}{\underline{e}}_{3}⊗{\underline{e}}_{3}$ , yielding:

$$\begin{array}{cc} \lambda_{axial}^{tis}\left(t_{n},{\underline{e}}_{3}\right) & ={\left({\underline{e}}_{3}\cdot F^{tis,T}\left(t_{n}\right)\cdot F^{tis}\left(t_{n}\right)\cdot {\underline{e}}_{3}\right)}^{1/2}\\ \lambda_{transverse}^{tis}\left(t_{n},{\underline{e}}_{1}\right) & ={\left({\underline{e}}_{1}\cdot F^{tis,T}\left(t_{n}\right)\cdot F^{tis}\left(t_{n}\right)\cdot {\underline{e}}_{1}\right)}^{1/2} \end{array}$$ (45)

where the base vectors ${\underline{e}}_{1}$ and ${\underline{e}}_{3}$ are those depicted in Figure 2. We are also particularly interested in the stretches of the collagen bundle phases, reading as:

$$\lambda_{axial}^{col}\left(t_{n},{\underline{e}}_{r}\left(t_{0}\right)\right)={\left({\underline{e}}_{r}\left(t_{0}\right)\cdot f^{col,T}\left(t_{n}\right)\cdot f^{col}\left(t_{n}\right)\cdot {\underline{e}}_{r}\left(t_{0}\right)\right)}^{1/2}$$ (46)

where the base vector ${\underline{e}}_{r}$ is also seen in Figure 2.

## 4 Micromechanical Modeling Results

### 4.1 Sensitivity Analysis: Uniaxial Stress-stretch Behavior Governed by Collagen Bundle Properties

**FIGURE 5:**
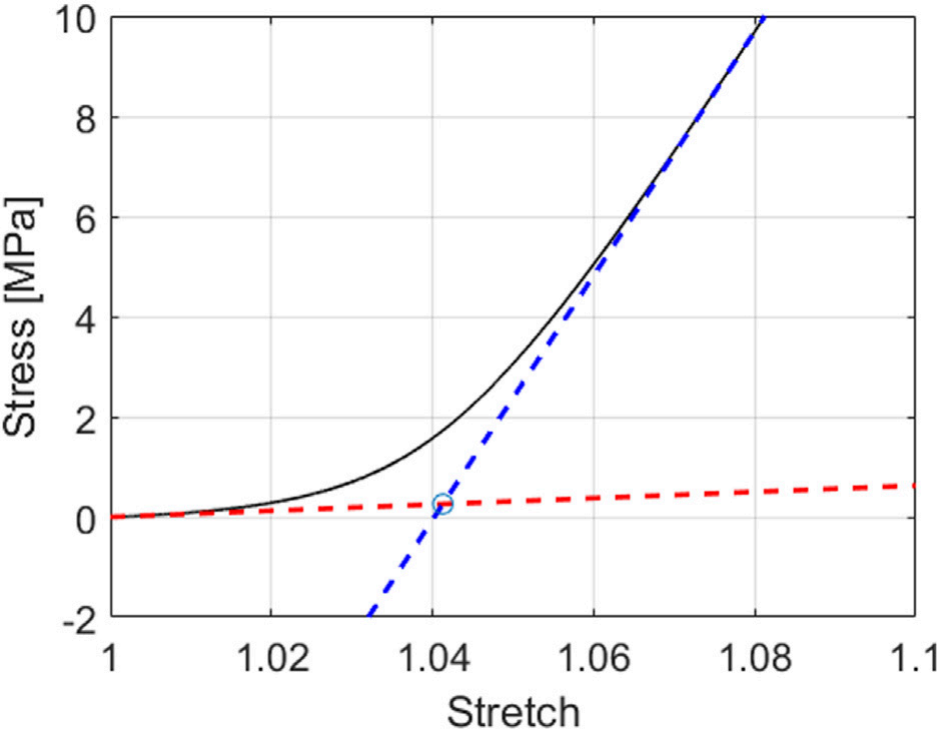
Model-predicted stress-stretch curve for tendinous tissue subjected to uniaxial tensile stress state; for *E*_*col*_ = 500 MPa, *E*_*m*_ = *E*_*μ*_ = 2.5 MPa, $\theta_{col}^{ini}=27.5{\;}^{{}^{\circ}}$ , $f_{col}^{fas}=0.6$ , and $f_{fas}^{tis}=0.95$ ; with indication of metrics used in sensitivity analysis: initial slope (*see* red tangent), final slope (*see* blue tangent), tangent intersection point (*see* circular mark) - the coordinates of the latter quantify intersection stress and intersection stretch.

First, the algorithm of Section 3.2 was used for analyzing the sensitivity of the micromechanical model responses to changes in three model input quantities associated with the collagen bundle phases: the Young’s modulus *E*_*col*_, the initial crimping angle $\theta_{col}^{fas}\left(t=0\right)=\theta_{col}^{ini}$ - here we consider the same initial value for all collagen bundle phases - and for the volume fraction $f_{col}^{fas}$ . Three different values for *E*_*col*_, seven different values for $\theta_{col}^{ini}$ , and four different values for $f_{col}^{fas}$ have been chosen, *see* Table 2. These values cover the ranges of experimental data described in Section 3.1. Correspondingly, *N*_*sim*_ = 3 × 7 × 4 = 84 micromechanical simulations based on the algorithm of Section 2.3 were performed. Guided by stress-stretch experiments which are customary in soft tissue research (*see* Section 4.2 for further details), the aforementioned simulations concerned uniaxial stress states, and corresponding stretches in the longitudinal tissue direction. Focusing on fiber re-orientation rather than fiber volume changes, a limited interval of stresses was investigated, ranging from 0 to 10 MPa (*see* Section 5 for a more detailed discussion on this aspect). This nonlinear behavior was quantified in terms of initial and final tangents. Thereby, the initial tangent was defined as the average, over the first 25 kPa of stress, of the tangents to the uniaxial stress-stretch curve; and the final tangent was defined as the average, over the last 500 kPa of stress, of the tangents to the uniaxial stress-stretch curve. Moreover, the coordinates of their intersection point in the stress-stretch plane are referred to as intersection stress and intersection stretch, respectively. Based on these quantities, and on the evolving crimping angle, the following metrics were used to analyze the model response, *see* also Figure 5:

• the slope of the initial tangent, referred to as initial slope;
• the slope of the final tangent, referred to as final slope;
• the intersection stretch;
• the intersection stress;
• the straightening angle, defined as the difference between the values for the crimping angle at the beginning and the end of each of the 84 simulations.

**TABLE 2:** Parameters studied in the sensitivity analysis.

Parameter	Minimum value	Step value	Maximum value
***E***_***col***_ **[GPa]**	0.3	0.2	0.7
$\theta_{col}^{ini}$ **[** **°** **]**	15	5	45
$f_{col}^{fas}$	0.6	0.1	0.9

**FIGURE 6:**
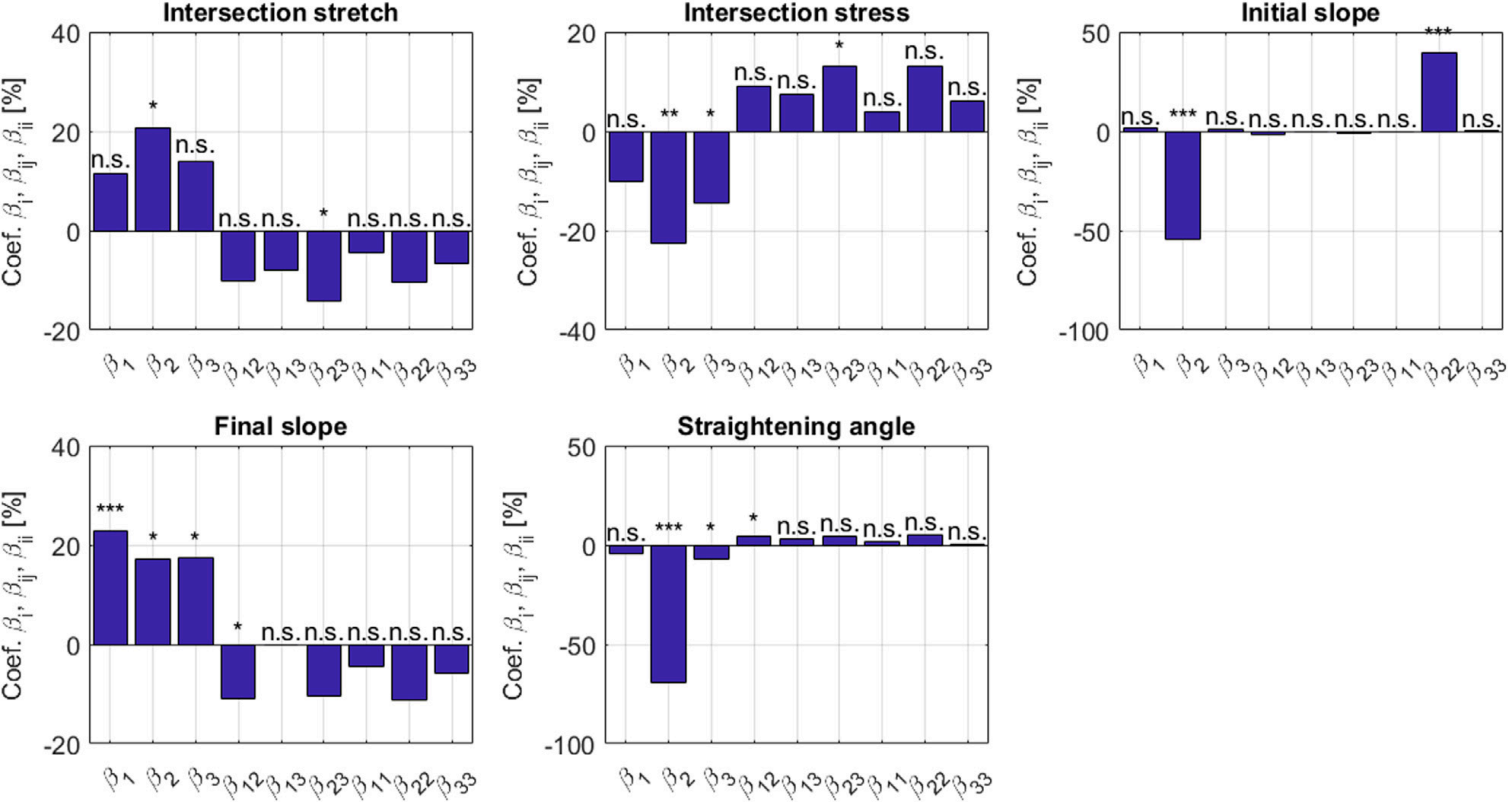
Sensitivity coefficients *β*_*i*_, *β*_*ij*_, *β*_*ii*_ according to Eqs 47, 48, with the indices 1, 2, and 3 relating to modulus, crimping angle, and volume fraction, respectively; determined for five metrics *Y* associated with the uniaxial stress-stretch behavior on the level of the tendinous tissue. The stars denote the significance level of the coefficients: three (resp. two and one) stars for a *p*-value below 10^–3^ (resp. 10^–2^ and 0.05); *n*.*s*. stands for non significant.

A metric-specific hypersurface over the hyperplane spanned by the normalized parameters

$$\begin{array}{cc} x_{1} & =\frac{E_{col}-E_{col}^{MIN}}{E_{col}^{MAX}-E_{col}^{MIN}}\\ x_{2} & =\frac{\theta_{col}^{ini}-\theta_{col}^{ini,MIN}}{\theta_{col}^{ini,MAX}-\theta_{col}^{ini,MIN}}\\ x_{3} & =\frac{f_{col}^{fas}-f_{col}^{fas,MIN}}{f_{col}^{fas,MAX}-f_{col}^{fas,MIN}} \end{array}$$ (47)

was fitted by means of second-order polynomial with first-order interactions, reading mathematically as (Tinsson, 2011):

$$Y=\beta_{0}+\overset{3}{\underset{i=1}{\sum }}\beta_{i}x_{i}+\overset{3}{\underset{i,j=1,j>i}{\sum }}\beta_{ij}x_{i}x_{j}+\overset{3}{\underset{i=1}{\sum }}\beta_{ii}x_{i}^{2}$$ (48)

where *x*_*i*_, with *i* = 1, 2, 3, refers to the normalized parameters according to Eq. 47, *Y* is one of the five previously cited output metrics of the model, *β*_0_ covers the portion of the metric *Y* which is not depending on *x*_1_, *x*_2_, and/or *x*_3_; and where *β*_*i*_, *β*_*ij*_, and *β*_*ii*_, with *i*, *j* = 1, 2, 3, reflect the sensitivity of the model with respect to the parameters *x*_1_, *x*_2_, and *x*_3_. It turns out that the polynomial expression Eq. 48 represents the micromechanical model results very well, quantified by a coefficient of determination amounting to 99% for all the tested metrics. The corresponding coefficients *β*_*i*_, *β*_*ij*_, *β*_*ii*_, with *i* = 1, 2, 3, are depicted in Figure 6, where three stars indicate a significant contribution of the corresponding normalized parameter on the micromechanical model result, as tested by a Student’s *t* test with (*N*_*sim*_ − 3) parameters. The following observations are noteworthy:

1. Intersection stress and intersection strain are very sensitive to the initial crimping angle, while the effect of bundle volume fractions is much less pronounced, and the bundle elasticity remains even insignificant in this context.
2. A similar situation is encountered with the initial slope, while the final slope, profoundly driven by the bundle modulus, shows some dependence on the bundle volume fraction and on the initial crimping angle.
3. The straightening angle is virtually exclusively driven by the initial crimping angle.

### 4.2 Comparison to Stress-stretch Experiments

**FIGURE 7:**
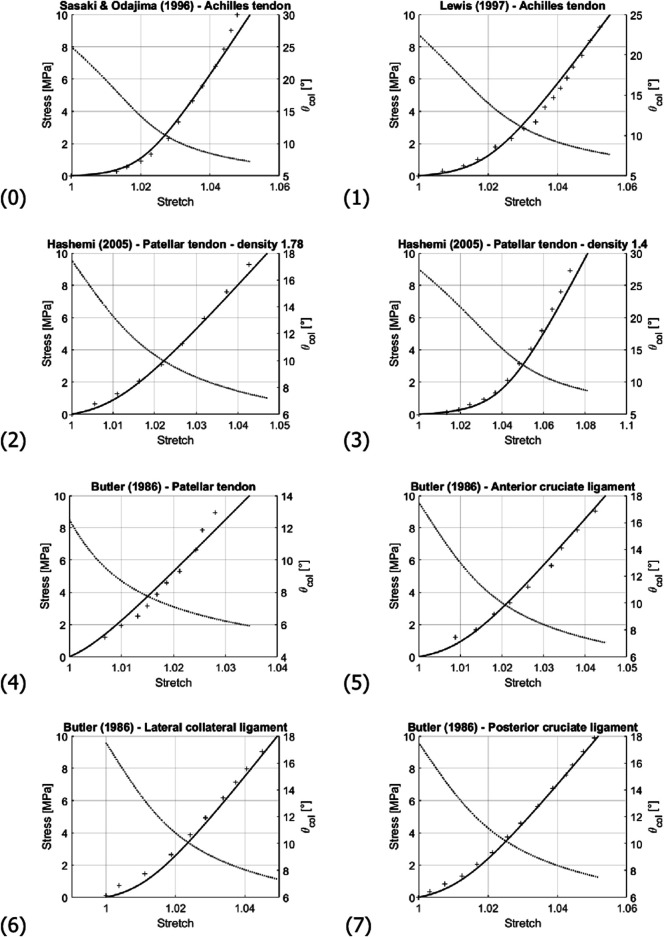
Comparison of the experimentally-measured (crosses) and predicted (solid line) stress-stretch curve; and evolution of the collagen fibril inclination angle *θ*_*col*_ (dashed line). Numbering of the subfigures refers to Table 3. Note that stress and angle values are labelled at the left and right sides of the diagrams, respectively.

**FIGURE 8:**
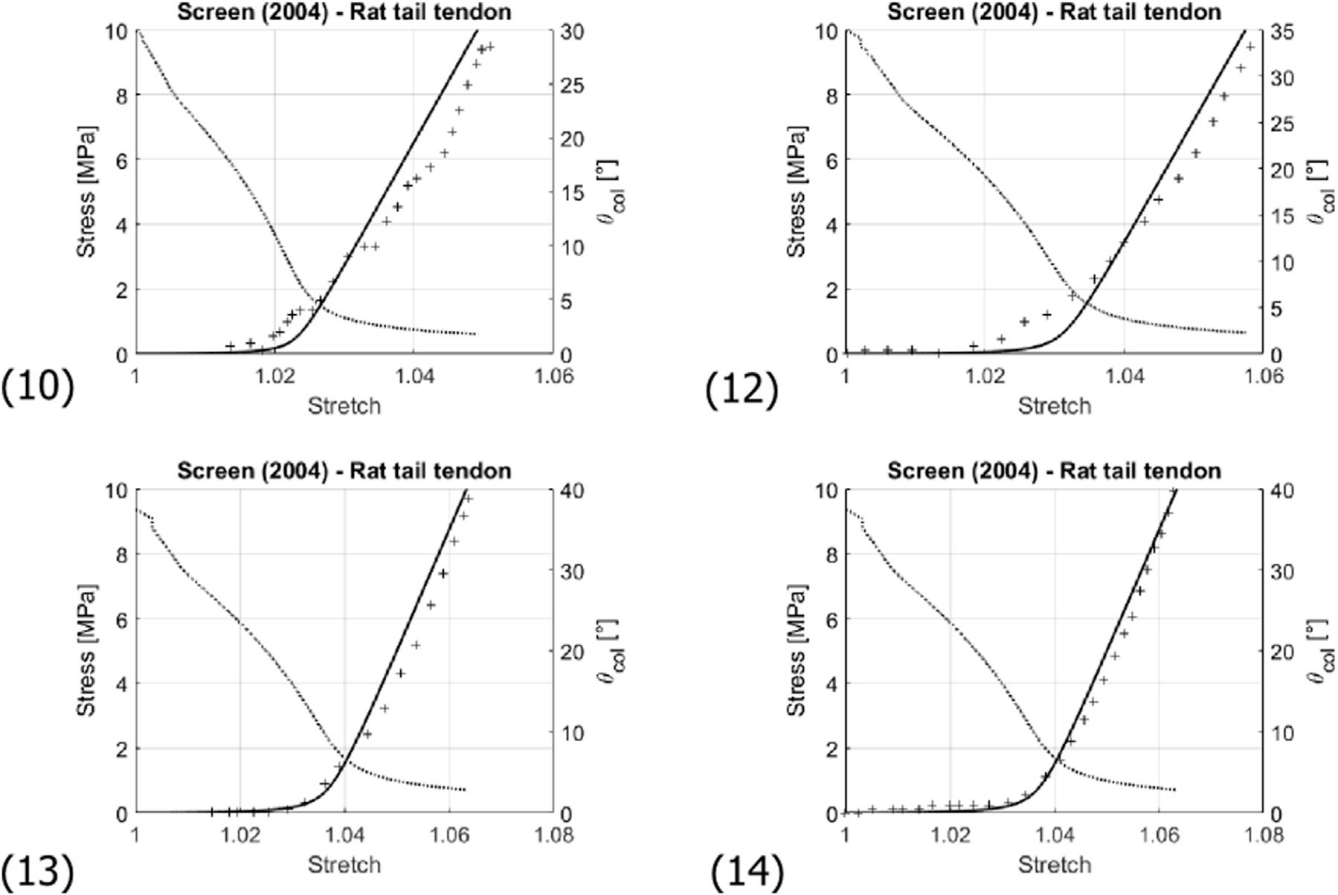
Comparison of the experimentally-measured (crosses) and predicted (solid line) stress-stretch curve of fascicles of rat tail tendons; and evolution of the collagen fibril inclination angle *θ*_*col*_ (dashed line). Numbering of the subfigures refers to Table 3. Note that stress and angle values are labelled at the left and right sides of the diagrams, respectively.

Next, micromechanical model results are directly compared to the experimental data stemming from uniaxial stress-stretch tests on bovine, human, and murine tendons (*see* Table 3 as well as Figures 7,8). These tests had been performed *in vitro* at (high) strain rates of 10–100%/s, suggesting a subordinate role of viscous effects. Therefore, bundle-related parameters within the ranges given in Table 3 were adopted, targeting high coefficients of determination *R* ^2^ and small residual errors *ϵ*:

$$R^{2}=1-\frac{\int_{\Sigma =0}^{\Sigma_{max}}{\left[\Lambda_{mod}\left(\Sigma \right)-\Lambda_{exp}\left(\Sigma \right)\right]}^{2}d\Sigma }{\int_{\Sigma =0}^{\Sigma_{max}}{\left[\Lambda_{exp}\left(\Sigma \right)-{\bar{\Lambda }}_{exp}\right]}^{2}d\Sigma };$$ (49)

$$\epsilon =\frac{1}{\Sigma_{max}}\int_{\Sigma =0}^{\Sigma_{max}}\left|\Lambda_{mod}\left(\Sigma \right)-\Lambda_{exp}\left(\Sigma \right)\right|d\Sigma ;$$ (50)

whereby the integrals are computed on the whole stress history, $\Lambda_{mod}\left(\Sigma \right)$ and $\Lambda_{exp}\left(\Sigma \right)$ being the longitudinal stretches corresponding to a uniaxial stress Σ, respectively computed by the model or reached experimentally, and ${\bar{\Lambda }}_{exp}$ being the average, over the entire load history, of the experimentally measured stretches. We note that the stress-stretch curves reported by Screen et al. (2004b) show an unusual start of the so-called toe region in the stress-stretch curve, involving decreasing slopes at small strains. Such effects cannot be explained by fiber re-orientation, and may rather result from instrumental challenges. We abstain from a deeper analysis of this issue, and simply start considering corresponding experimental data whenever a minimum slope has been reached in the toe region.

**TABLE 3:** Collection of experimental references for stretch-stress data given in Figures 7, 8, together with optimized values for initial fiber orientation and collagen volume fraction, in order to reach the coefficients of determination and the residual errors in the last two columns; all other model input data are found in Table 4.

	Reference	Tendon	Species	$\theta_{col}^{ini}$	$f_{col}^{fas}$	*R* ^2^	*ɛ* (%)
0	Sasaki and Odajima (1996b)	Achilles	bovine	25	0.75	0.951	0.20
1	Lewis and Shaw (1997)	Achilles	young human	22.5	0.675	0.983	0.13
2	Hashemi et al. (2005)	PT	young human	17.5	0.65	0.990	0.09
3	Hashemi et al. (2005)	PT	young human	27.5	0.6	0.984	0.22
4	Butler et al. (1986)	PT	young human	12.5	0.725	0.894	0.16
5	Butler et al. (1986)	ACL	young human	17.5	0.675	0.990	0.08
6	Butler et al. (1986)	LCL	young human	17.5	0.625	0.979	0.14
7	Butler et al. (1986)	PCL	young human	17.5	0.6	0.993	0.09
10	Screen et al. (2004b)	tail	Wistar rats	30	0.8	0.914	0.29
12	Screen et al. (2004b)	tail	Wistar rats	35	0.8	0.770	0.57
13	Screen et al. (2004b)	tail	Wistar rats	37.5	0.8	0.738	0.55
14	Screen et al. (2004b)	tail	Wistar rats	32.5	0.725	0.788	0.53

PT, patellar tendon; ACL, anterior cruciate ligament; LCL, lateral colateral ligament; PCL, posterior cruciate ligament.

**TABLE 4:** Input values for the micromechanical model.

Angles		
$\theta_{col}^{fas}$ [°]	[15–45]	*see* Figure 4
$\theta_{col}^{tis}$ [°]	0	Kastelic et al. (1978)
$\phi_{col}^{fas}$ [°]	0, 90, 180, 270	De Campos Vidal (2003); Kalson et al. (2012)
$\phi_{col}^{tis}$ [°]	0	Kastelic et al. (1978)
**Volume fractions**		
$f_{col}^{fas}$	[0.6–0.9]	*see* Table 1
$f_{col}^{tis}$	0.95	Patterson-Kane et al. (2012)
**Mechanical parameters**		
*E*_*col*_ [MPa]	500	Sasaki and Odajima (1996b)
*E*_*m*_ [MPa]	2.5	Franke et al. (2007)
*E*_*μ*_ [MPa]	2.5	Franke et al. (2007)
*ν*_*col*_	0.34	Cusack and Miller (1979)
*ν*_*m*_	0.34	Cusack and Miller (1979); Urayama et al. (1993); Li et al. (1993)

For all simulations, the modulus value *E*_*col*_ of Sasaki and Odajima (1996a), amounting to 500 MPa, was taken as reference, being able to represent all considered experimental data in a satisfactory manner, *see* Table 3 and Figure 7. Optimization of the values taken for *f*_*col*_^*fas*^ and $\theta_{col}^{ini}$ was guided by the sensitivity analysis of Section 4.1, leading to the results of Table 3. As observed in the experiments of (Abrahams, 1967; Hansen et al., 2002), the pseudo-linear portion of the stress-stretch curve is associated with the crimping angle approaching an almost constant level.

## 5 Discussion and Conclusion

In this study, we have traced back the non-linear behavior of soft tissues in general, and more specifically of tendons, to normal and shear deformations as well as to rigid body motions (rotations) of straight, elongated, long, and stiff fibers (representing collagen bundles in the case of tendons) embedded in soft matrices.

The corresponding micromechanical representation directly reflects the extreme lengths of the 50 μm thick collagen bundles, spanning over several millimeters. This was evidenced by a series of transmission electron micrographs (TEM) (Provenzano and Vanderby, 2006; Craig et al., 1989; Parry and Craig, 1984; Svensson et al., 2017) showing tens of thousands of bundles over test domains spanning several milimeters, without any indication of ending bundles or bundle joints. Also the mechanical role of the gel-type matrix, the deformation of which is essential for the behavior of the overall fascicle and tendinous tissue-related RVEs, is consistent with experimental observations: Inhibiting the binding of matrix-proteins like decorin to the collagen fibrils changes the stress-strain behaviors by leaving more deformational freedom to the fibers, which eventually results in tendon lengthening with respect to untreated control tissues (Caprise et al., 2001). Our model also accounts for the crimped nature of the bundles; however, in a simplified manner: the collagen bundle phases are not wavy, but straight - still, they are oriented in different direction *in space*: this is consistent with the helical, rather than a planar, nature of crimping, as seen from the microcopic observations of (De Campos Vidal, 2003). As mentioned before, the omission of actual curvature modeling is consistent with the low bending stiffness of the bundles (Yang et al., 2008b,a): what counts upon decrimping is the recruitment of stretching stiffness in combination with matrix shearing - a mechanism which is explicitly considered by our model. This renders our model as a prime candidate for making larger scale finite element models more realistic and reliable, in the same way as already shown for arterial tissue in greater detail (Bianchi et al., 2020).

**FIGURE 9:**
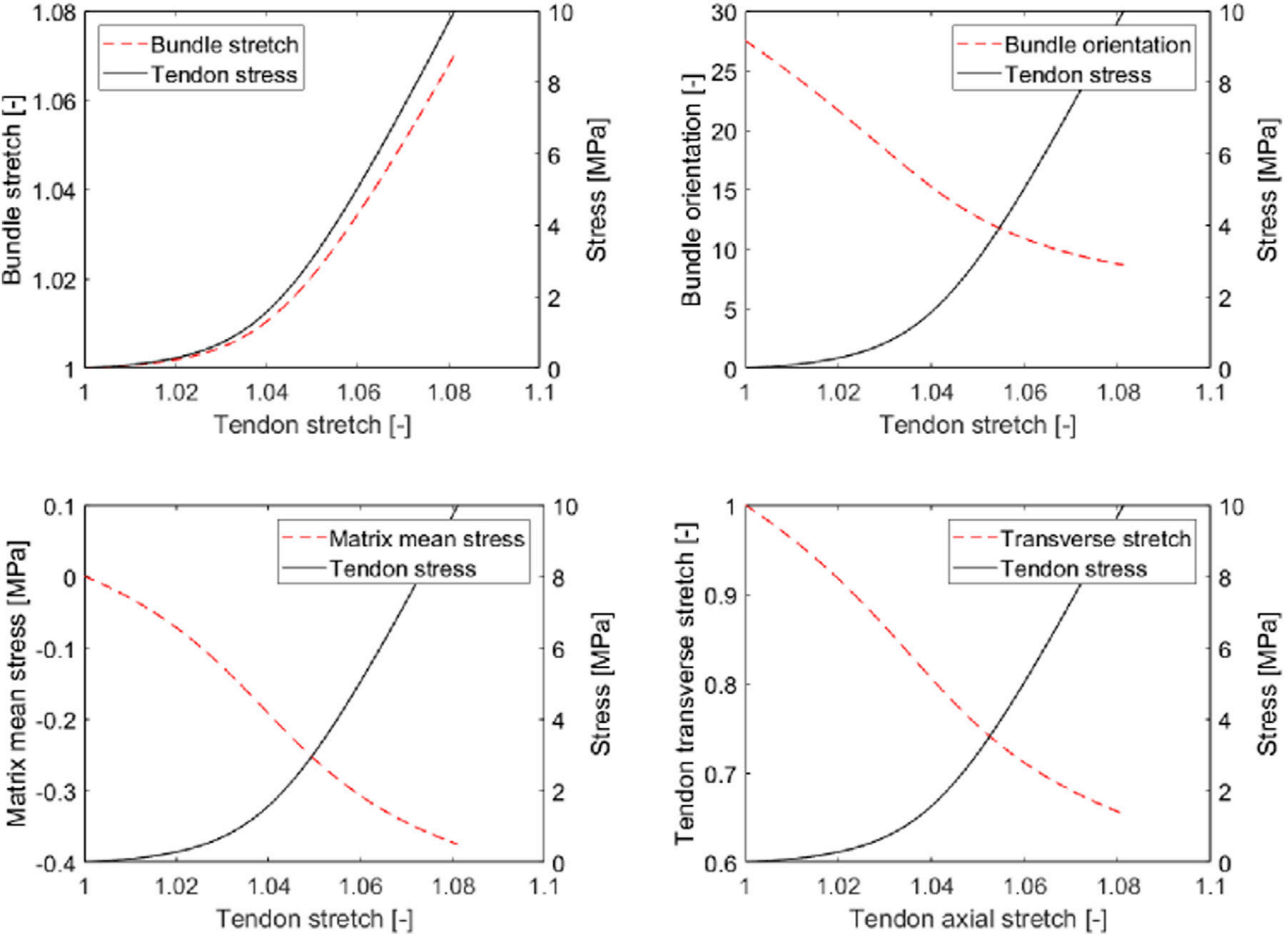
Micromechanical model predictions for tendinous tissue subjected to uniaxial tension: axial stretch in collagen bundle **(top left)**, latitudinal angle of collagen bundle **(top right)**, mean stress in interfascicular matrix **(bottom left)**, and transverse stretch in tendinous tissue **(bottom right)**, as functions of axial stretch in tendinous tissue.

It is very illustrative to study the model-predicted microscopic stresses prevailing in the collagen bundles and in the matrix inbetween, see Figure 9: Under uniaxial macroscopic tensile loading, all the bundle phases are loaded in tension, while the matrix undergoes compression. This fits perfectly with the experimental observation of fluid being pressed out of tendinous tissue upon macroscopic uniaxial tensile load (Lanir et al., 1988; Hannafin and Arnoczky, 1994; Thornton et al., 2001). We also observe that the fibrillar stretch is much smaller than the tendon stretch. Accordingly, toe region-related stretching is microstructurally accomodated by rigid body movements (rotations) of the fibers, a mechanism already described in the landmark work of Diamant et al. (1972). In this context, we also note that the order of magnitude of model-predicted stretches at the collagen bundle level agrees well with the measurements of Screen et al. (2002, 2004a). This microstructurally modeled mechanical behavior naturally avoids unphysical Poisson effects including even auxetic behavior, as they are known from traditional hyperelastic modeling (Skacel and Bursa, 2016; Volokh, 2017; Skacel and Bursa, 2019). As a remedy, Fereidoonnezhad et al. (2020) introduced a formulation involving “matrix strain stiffening.” By comparison, our model does not introduce any fiber or matrix strain stiffening, but constant hypoelastic values in accordance with experimental data characterizing the microstructural components of soft tissue. In more detail, instead of enforcing increased load bearing of the matrix, and primarily so throughout the lower stretch regime, our model reveals that already then, non-negligible fiber stretches contribute to the overall tissue response, *see* also Figure 9 (top left).

Still, our model exhibits several limitations. This first concerns the fixation of the volume fractions used in the simulations presented herein. This simplification may indeed restrict the predictive potential of our micromechanical model, as follows from the following deliberations:

• at low macroscopic stretch, the fibers re-orient and virtually do not stretch; hence they hardly change their volume;
• at high stretch, the fibers are elongated, and their volume does change.

Such big differences in volume change between low and high macroscopic stretch are not expected for the matrix. Hence, remarkably changing fiber volume fractions may be indeed expected; and such probably higher fiber volume fractions at higher stretches are consistent with our model underestimation for stresses reaching values between 5 and 10 MPa in several of the prediction curves (see curves (2), (3), and (4) in Figure 7). The significance of considering the actually load-dependent volume fractions would clearly increase when modeling the tissue behavior under higher stress levels than those seen in Figures 7, 8. For such stress states, it would hence be advisable to update, not only the fiber orientations, but also the fiber volume fractions after every load step.

The second limitation of the model relates to its restriction to elasticity, leaving out classical mechanical properties, in particular viscous, plastic, and damage effects (Puxkandl et al., 2002; Weiss et al., 2002; Haut and Haut, 1997). Potential inclusion of viscous and viscoelastic effects into the herein presented model may start with the extension of the hypoelastic constitutive Eq. 20 towards suitable hereditary integrals similar of those proposed by Boltzmann (1874) or Volterra (1909) for the small strain regime (Gurtin and Sternberg, 1962). At higher stress levels, extensions towards so-called non-linear viscoelasticity may be necessary (Pipkin and Rogers, 1968; Johnson et al., 1996). As concerns the upscaling of such a viscoelastic phase behavior, one may take again inspiration from small strain homogenization theory (Laws and McLaughlin, 1978; Eberhardsteiner et al., 2014). In more detail, Laplace-Carson transforms of the aforementioned hereditary integrals may deliver sequences of formally (hypo-)elastic problems to which the strategy of Section 2.3 remains fully applicable. Potential inclusion of plastic effects into the herein presented model may start with the extension of the hypoelastic constitutive Eq. 20 towards eigenstrain rates the evolution of which obeys suitable plastic flow rules. Thereafter, eigenstrain rate upscaling may follow from extension of respective homogenization theories developed for the small strain regime (Dvorak, 1992; Pichler and Hellmich, 2010; Königsberger et al., 2020), thereby extending recent developments for hard tissues (Fritsch et al., 2009; Blanchard et al., 2016; Morin et al., 2017) towards the realm of soft tissues.

The third limitation concerns the non-coverage of multiphysics effects, such as mechano-electrochemical couplings including osmotic pressures (Wilson et al., 2005; Masic et al., 2015), leading to phenomena which have been described as “inverse poroelasticity” (Ehret et al., 2017). Again, we think that eigenstrain upscaling appears as an interesting option to consider such effects as well.

From a micromorphological viewpoint, one may also ask whether the relatively simple micromechanical representation sketched in Figure 3 may be another limitation of the present model. Diagram (10) and (12) of Figure 8 might indeed indicate a situation where more than one fiber recruitment process takes place; hence, the existence of more than one prominent initial latitudinal fiber angle. However, these diagrams might also simply reflect experimental uncertainties rather than model limitations.

Conclusively, we presented a novel micromechanical model providing a natural access to the non-affine, non-auxetic, microstructurally driven elastic behavior of tendon; resting on hypoelastic phase properties combined with an objective kinematics, giving access to proper strain-to-strain and strain-to-spin relations across the hierarchical organization of tendons. In this context, our model may well be seen as an interesting, computationally efficient, complement to the growing number of fiber network models proposed for soft tissues (Chandran and Barocas, 2006; Stylianopoulos and Barocas, 2007; Cyron et al., 2013; Picu et al., 2018). With these models, we share the explicit consideration of non-affine fiber re-orientations leading to pronounced lateral contractions under uniaxial tensile loading. Still, our present approach goes beyond the scope of the aforementioned network models when it comes to the explicit introduction of the mechanical behavior of the gel-type matrices. Most remarkably, model-predicted hydrostatic pressures prevailing in the interfascicular matrix (also known as the endotenon, which hosts vascular cells according to (Kannus, 2000; Godinho et al., 2017)) exhibit a magnitude which stimulates a variety of biological cells in the musculo-skeletal system; *see* (Scheiner et al., 2016) for a compilation of various experimental sources and data; and the stimulatory effect of hydrostatic pressures in the tens of kilopascals range has been shown explicitly for endothelial vascular cells as well (Ohashi et al., 2007). This opens perspectives for extending the current fiber-matrix interaction model towards the realm of tissue remodeling, in a way already realized for bone (Pastrama et al., 2018).

## Data Availability

The raw data supporting the conclusions of this article will be made available by the authors, without undue reservation.
