# Supplementary material for: Fiber Rearrangement and Matrix Compression in Soft Tissues: Multiscale Hypoelasticity and Application to Tendon
Source: Front Bioeng Biotechnol. 2021 Oct 12;9:725047. doi: 10.3389/fbioe.2021.725047 (PMC8546211; doi:10.3389/fbioe.2021.725047)
Supplement: Supplementary file 1 [file DataSheet1.pdf]

Fiber rearrangement and matrix compression in soft tissues: Multiscale hypoelasticity and application to tendon - Supplementary material

Claire Morin [1,*], **Christian Hellmich** [2], **Zeineb Nejim** [1] **and Stéphane Avril** [1,2]

[1] *Mines Saint-Etienne, Univ. Lyon, Univ. Jean Monnet, INSERM, U1059 Sainbiose, Centre CIS, F – 42023 Saint-Etienne, France*

[2] *TU Wien - Institut for Mechanics of Materials and Structures. Vienna University of Technology, 1040 Vienna, Austria*

Correspondence*:
Claire Morin
claire.morin@mines-stetienne.fr

1 NOMENCLATURE

NOMENCLATURE

$\mathbb{A}_m$	fourth-order strain rate concentration tensor of matrix phase
$\mathbb{A}_m^\infty$	matrix-inclusion problem-related strain rate concentration tensor of matrix phase
$\mathbb{A}_{fas}^{tis}$	fourth-order strain rate concentration tensor of fascicle phase in the tendinous tissue RVE
$\mathbb{A}_m^{tis}$	fourth-order strain rate concentration tensor of matrix phase in the tendinous tissue RVE
$\mathbb{A}_{col,r}^{fas}$	fourth-order strain rate concentration tensor of r-th collagen fiber phase in the fascicle RVE
$\mathbb{A}_\mu^{fas}$	fourth-order strain rate concentration tensor of matrix phase in the fascicle RVE
$\mathbb{A}_r$	fourth-order strain rate concentration tensor of the r-th fiber phase
$\mathbb{A}_r^\infty$	matrix-inclusion problem-related strain rate concentration tensor of the r-th fiber phase
$\mathbb{C}_{fas}$	homogenized fourth-order stiffness tensor of the fascicle
$\mathbb{C}_{fas}^{tis}$	fourth-order hypoelasticity tensor of the fascicle phase within the tendinous tissue RVE
$\mathbb{C}_m^{tis}$	fourth-order hypoelasticity tensor of the matrix phase within the tendinous tissue RVE
$\mathbb{C}_{col}^{fas}$	fourth-order hypoelasticity tensor of the collagen bundle phase within the fascicle RVE
$\mathbb{C}_\mu^{fas}$	fourth-order hypoelasticity tensor of the matrix phase within the fascicle RVE
$\mathbb{C}_{hom}$	homogenized fourth-order stiffness tensor
$\mathbb{C}_m$	fourth-order hypoelasticity tensor of matrix phase
$\mathbb{C}_r$	fourth-order hypoelasticity tensor of the r-th fiber phase
$\mathbb{C}_{tis}$	homogenized fourth-order stiffness tensor of the tendinous tissue
d	characteristic size of the inhomogeneities within the RVE
$\mathscr{D}$	dissipation
d_{col}	characteristic diameter of the collagen bundles
d_{fas}	characteristic diameter of the fascicles
$\boldsymbol{D}$	(homogeneous) macroscopic second-order strain rate tensor
$\boldsymbol{D}^{fas}$	(homogeneous) macroscopic second-order strain rate tensor applied on the fascicle RVE
$\boldsymbol{D}^{tis}$	(homogeneous) macroscopic second-order strain rate tensor applied on the tendinous tissue RVE
$\boldsymbol{D}^{tis,est}$	estimated (homogeneous) macroscopic second-order strain rate tensor for stress-imposed loading
$\boldsymbol{d}$	microscopic second-order strain rate tensor
$\boldsymbol{d}_m$	microscopic second-order strain rate tensor averaged over the matrix phase

$\boldsymbol{d}_r$	microscopic second-order strain rate tensor averaged over the r-th fiber phase
$\boldsymbol{d}_{fas}^{tis}$	second-order strain rate tensor averaged over the fascicle phase in an RVE of tendinous tissue
$\boldsymbol{d}_{m}^{tis}$	second-order strain rate tensor averaged over the matrix phase in an RVE of tendinous tissue
$\boldsymbol{d}_{col,r}^{fas}$	second-order strain rate tensor averaged over the r-th collagen bundle phase in a fascicle-related RVE
$\boldsymbol{d}_{\mu}^{fas}$	second-order strain rate tensor averaged over the matrix phase in a fascicle-related RVE
$\underline{e}_r, \underline{e}_\theta, \underline{e}_\phi$	spherical base vectors
E	Young's modulus
E_{col}	Young's modulus of the collagen bundle phase
$E_\mu = E_m$	Young's modulus of the matrix phase
f_m	volume fraction of the matrix phase
f_r	volume fraction of the r-th fiber phase
f_{fas}^{tis}	volume fraction of the fascicle phase in a RVE of tendinous tissue
f_{m}^{tis}	volume fraction of the matrix phase in a RVE of tendinous tissue
$f_{col,r}^{fas}$	volume fraction of the r-th collagen bundle phase in a fascicle-related RVE
f_{μ}^{fas}	volume fraction of the matrix phase in a fascicle-related RVE
$\mathcal{G}_\rho$	Gibbs free energy per unit mass
$\mathbb{I}$	fourth-order identity tensor
$\mathbb{J}$	spherical part of the fourth-order identity tensor
$\mathbb{K}$	deviatoric part of the fourth-order identity tensor
k	bulk modulus
$\mathscr{L}$	characteristic length of the structure or of its loading
ℓ	characteristic length of the RVE
ℓ_{fas}	characteristic length of a fascicle-related RVE
ℓ_{tis}	characteristic length of an RVE of tendinous tissue
N_f	number of fiber phases in an RVE
N_{sim}	number of simulations for sensitivity analyses
$\underline{n}$	unit normal outward vector at microscopic scale
N_t	number of time points
$\mathcal{P}^{ext}$	power density of external forces acting on the RVE
$\mathbb{P}_r$	fourth-order Hill tensor of phase r
R^2	coefficient of determination
$\mathbb{R}_r$	fourth-order strain rate-to-spin concentration tensor of the r-th fiber phase
$\mathbb{R}_r^\infty$	matrix-inhomogeneity problem-related strain rate-to-spin concentration tensor of the r-th fiber phase
$\mathbb{R}_r^{Esh}$	fourth-order Eshelby-type tensor relating eigenstrain rate in an ellipsoidal inclusion to inclusion spin
$\mathbb{R}_{fas}^{tis}$	fourth-order strain rate-to-spin concentration tensor of the fascicle phase in an RVE of tendinous tissue
$\mathbb{R}_{col,r}^{fas}$	fourth-order strain rate-to-spin concentration tensor of the r-th collagen bundle phase in a fascicle-related RVE
RVE	representative volume element
SHG	second harmonic generation
$\mathbb{S}_r^{Esh}$	fourth-order Eshelby tensor relating eigenstrain rate in an ellipsoidal inclusion to the total strain rate in that inclusion
t_n	time point

$\underline{t}$	(microscopic) traction vector applied on the boundary of the RVE
TEM	transmission electron microscopy
$\underline{v}$	microscopic velocity field
$\underline{x}$	position vector of microscopic material points
$\underline{X}$	position vector of macroscopic material points
x_i	normalized parameter value in the sensitivity analysis
Y	output metric of the sensitivity analysis model
$\beta_i, \beta_{ij}, \beta_{ii}$	coefficients of the fitting polynomial used for the sensitivity analysis
Δt	time interval
ϵ	residual error
θ	spherical (co-latitudinal) coordinate
θ_{fas}^{tis}	spherical (co-latitudinal) coordinate of the fascicle in the tendinous tissue RVE
$\theta_{col,r}^{fas}$	spherical (co-latitudinal) coordinate of the collagen bundle in the fascicle RVE
$\bar{\Lambda}_{exp}(\Sigma)$	average, over the entire load history, of experimentally measured stretches
$\Lambda_{exp}(\Sigma)$	experimentally observed longitudinal stretch arising from a uniaxial stress prescribed in longitudinal direction
$\Lambda_{mod}(\Sigma)$	model-predicted longitudinal stretch corresponding to a uniaxial stress prescribed in longitudinal direction
μ	shear modulus
ν	Poisson's ratio
ν_m	Poisson's ratio of the matrix phase
ρ	microscopic mass density
$\boldsymbol{\Sigma}$	macroscopic second-order Cauchy stress tensor
$\boldsymbol{\Sigma}^{tis}$	(macroscopic) second-order Cauchy stress tensor of the tendinous tissue
Σ	uniaxial macroscopic stress applied in the longitudinal direction
$\boldsymbol{\sigma}$	microscopic second-order Cauchy stress tensor
$\boldsymbol{\sigma}_m$	microscopic second-order Cauchy stress tensor averaged over the matrix phase
$\boldsymbol{\sigma}_r$	microscopic second-order Cauchy stress tensor averaged over the r-th fiber phase
Σ_{max}	maximum uniaxial stress applied in the longitudinal direction
$\boldsymbol{\sigma}_{fas}^{tis}$	microscopic second-order Cauchy stress tensor averaged over the fascicle phase in an RVE of tendinous tissue
$\boldsymbol{\sigma}_{m}^{tis}$	microscopic second-order Cauchy stress tensor averaged over the matrix phase in an RVE of tendinous tissue
$\boldsymbol{\sigma}_{col,r}^{fas}$	microscopic second-order Cauchy stress tensor averaged over the r-th collagen bundle phase in a fascicle-related RVE
$\boldsymbol{\sigma}_{\mu}^{fas}$	microscopic second-order Cauchy stress tensor averaged over the matrix phase in a fascicle-related RVE
ϕ	spherical (longitudinal) coordinate (Euler angle)
$\phi_{col,r}^{fas}$	spherical (longitudinal) coordinate of the r-th collagen bundle within the fascicle RVE

Ω	volume of an RVE
Ω_m	volume of the matrix phase
Ω_r	volume of the r-th fiber phase
$\partial\Omega$	external boundary of an RVE
$\boldsymbol{\omega}$	microscopic second-order spin tensor
$\boldsymbol{\omega}_m$	microscopic second-order spin tensor averaged over the matrix phase
$\boldsymbol{\omega}_r$	microscopic second-order spin tensor averaged over the r-th fiber phase
$\boldsymbol{\omega}_{fas}^{tis}$	microscopic second-order spin tensor averaged over the fascicle phase in an RVE of tendinous tissue
$\boldsymbol{\omega}_{col,r}^{fas}$	microscopic second-order spin tensor averaged over the r-th collagen fiber phase in a fascicle-related RVE
Operators	
div	divergence operator
$\partial(.)/\partial\underline{X}$	macroscopic gradient operator
$\partial(.)/\partial\underline{x}$	microscopic gradient operator
$(.)^T$	transpose of tensorial quantity $(.)$
$\langle(.)\rangle$	spatial average of quantity $(.)$, over the RVE
$\|\|(.)\|\|$	(quadratic) norm of quantity $(.)$
$\dot{(.)}$	rate (temporal derivative) of quantity $(.)$
$(.)^{\triangle}$	objective derivative of quantity $(.)$
$\cdot$	first-order tensor contraction (inner product)
:	second-order tensor contraction
$\equiv$	identical to
Subscripts	
col	... of the collagen bundles
fas	... of the fascicles
m	... of the interfascicle matrix
μ	... of the interbundle matrix

2 MATLAB CODE

Main code:

```
%Incremental Approach
er=tol+1;
SIGMA = zeros(6,1);
for u=1:Nu-1

    % Macroscopic stress increment
    dS1 = S(u+1,:)-S(u,:);
    % Retrieving angles, stress at time t
    theta=stocktheta(u,:);
    phi=stockphi(u,:);
    theta2_u=stocktheta2{u}';
    phi2_u=stockphi2{u}';
    sigmam=stocksigmam{u};
    sigmai_i=stocksigmai_i{u};
    sigmam2_u=stocksigmam2{u};
    sigma2i_u=stocksigma2i_i{u};

    D_incr1 = 0;
    while er >tol
        % Computing the strain rate that generated the stress
            increment
        D_incr = inv(Chom1) * dS1'/deltat    ;

        %first Scale

        %DOWNSCALING the macroscopic strain rate
        [Chom,di,dm,wi,wm,theta_new,phi_new,A_fib,A_m,R_fib,
            Ci_help_fib,sumAinf,Ci_rot] = downscaling_multi_edited(n_1,
             D_incr,fi,Ci,Cm,I,S_i,A_i,phi,theta,deltat,n_move); %+n_2+
            n_3

        %CONSTITUTIVE : computing the constitutive response of each
            phase
        [sigmai_r_new,sigmam_new] = constitutive_multi(n_1,sigmai_i,
            sigmam,di,dm,Ci_rot,Cm,wi,wm,deltat);

        %Second Scale

        for i=1:n_1
        [di_rot] = voigt(rot1(phi(i),theta(i),cancel_voigt(di{i})));
        SIGMA2_rot = sigma_fiber{u,i};
        [SIGMA2] = voigt(rot1(phi(i),theta(i),cancel_voigt(SIGMA2_rot
            )));
```

```
46
47          %DOWNSCALING
48          [Chom2_new, d2i, d2m, w2i, w2m, theta2_new, phi2_new, A2_fib, A2_m,
49              R2_fib, C2i_help_fib, sumAinf2, C2i_rot] =
50              downscaling_multi_edited(n2_i, di_rot, f2i, C2i, C2m, I, S2_i,
51              A2_i, phi2, theta2, deltat, n2_move);
52          %CONSTITUTIVE
53          [sigmai_i2_new, sigmam2_new] = constitutive_multi(n2_i,
54              sigma2i_i, sigmam2, d2i, d2m, C2i_rot, C2m, w2i, w2m, deltat);
55          %UPSCALING
56          [Chom2] = upscaling_multi(n2_i, sigmai_i2_new, sigmam2_new,
57              C2i_rot, C2m, A2_fib, A2_m, f2i, R2_fib);
58          SIGMA2 = SIGMA2 + Chom2 * di_rot ;
59
60          % Rotation back to the global basis
61          [SIGMA2_rot]= voigt(rot1retour(phi(i),theta(i),cancel_voigt(
62              SIGMA2)));
63          [Chom2_rot]= compress(rot2retour(phi(i),theta(i),expand(Chom2
64              ))) ;
65          [sigmai_i2_new_rot]= voigt(rot1retour(phi(i),theta(i),
66              cancel_voigt(sigmai_i2_new{1})));
67
68          %Retrieving stress in each family and matrix at time t
69          stocktheta2{u+1,i}= theta2_new;
70          stockphi2{u+1,i}= phi2_new;
71          %Stocking Stress in each family and matrix at time t+dt
72          stocksigmam2{u+1,i}=sigmam2_new;
73          stocksigma2i_i{u+1,i}=sigmai_i2_new;
74          sigma_fiber{u+1,i}=SIGMA2_rot;
75          sigmai_i_from2{i}=SIGMA2_rot;
76          Ci_rot{i}=Chom2_rot;
77          Ci_new{i} = Chom2;
78          end
79
80  %         UPSCALING
81          SIGMA = SIGMA + Chom1 * D_incr ;
82          theta= theta_new;
83          phi=phi_new;
84
85          er=norm(S(u+1,:) - SIGMA')/norm(S(u+1,:));
86          D_incr1 = D_incr1 + D_incr;
87
88      end
89  er=tol+1;
90          F=(cancel_voigt(D_incr1)+eye(3))*F;
```

```
91            stocktheta(u+1,:)= theta_new';
92            stockphi(u+1,:)= phi_new;
93            stocksigmam{u+1}=sigmam_new;
94            stocksigmai_i{u+1}=sigmai_r_new;
95            sigma_matrix{u}=sigmam_new;
96
97        stockSIGMA(u+1,:)=SIGMA';
98        F_M(u,:) = (voigt(F))';
99        Ci = Ci_new;
100
101   end
```

102 **Downscaling function:**

```
103   function [Chom,di,dm,wi,wm,theta_n,phi_n,A_fib,A_m,R_fib,
104       Ci_multi_A_esh,sum_f_multi_A_esh,Ci_global] = downscaling_latex(n_i
105       ,D,fi,Ci,Cm,I,S_i,A_i,phi,theta,deltat)
106
107   % time t
108   for i=1:n_i
109   vector_e_theta(i,:) = [cos(theta(i)).* cos(phi(i)) ; cos(theta(i)).*
110       sin(phi(i));-sin(theta(i))];
111   vector_e_phi(i,:) =    [-sin(phi(i)) ; cos(phi(i));zeros(size(theta(i)
112       ))];
113   vector_e_r(i,:) =      [sin(theta(i)).* cos(phi(i)) ; sin(theta(i)).*
114       sin(phi(i));cos(theta(i))];
115   end
116
117
118   %computing homogeneous stiffness and concentration strain rate and
119       spin tensor
120   [Chom,A_fib,A_m,R_fib,Ci_multi_A_esh,sum_f_multi_A_esh,Ci_global]=
121       eshelby_multi(n_i,fi,Ci,Cm,I,vector_e_r,vector_e_phi,S_i,A_i);
122
123   %computing microscopic strain rate and spin
124   for i=1:n_i
125        di{i} =A_fib{i} *D;
126        dm=A_m*D;
127        wm=zeros(6,1);
128        wi{i} = R_fib{i}* D;
129   end
130
131   for i=1:n_i
132   %Update base vectors
133        Delta_er(i,:)=((cancel_voigt(di{i})+cancel_voigt_antisym(wi{i}))*
134            vector_e_r(i,:)')';
```

```
135     vector_e_r(i,:) = (vector_e_r(i,:) + Delta_er(i,:)*deltat) / norm
136       (vector_e_r(i,:) + Delta_er(i,:)*deltat);
137
138     Delta_etheta(i,:)=((cancel_voigt(di{i})+cancel_voigt_antisym(wi{i
139       }))*vector_e_theta(i,:)')';
140     vector_e_theta(i,:) = (vector_e_theta(i,:) + Delta_etheta(i,:)*
141       deltat) / norm(vector_e_theta(i,:) + Delta_etheta(i,:)*deltat);
142
143     Delta_ephi(i,:)=((cancel_voigt(di{i})+cancel_voigt_antisym(wi{i})
144       )*vector_e_phi(i,:)')';
145     vector_e_phi(i,:) = (vector_e_phi(i,:) + Delta_ephi(i,:)*deltat)
146       / norm(vector_e_phi(i,:) + Delta_ephi(i,:)*deltat);
147
148 %Update angles theta and phi
149     theta_n(i,:) = acos(vector_e_r(i,3))  ;
150     phi_n(i,:) = acos(vector_e_phi(i,2)) ;
151     if vector_e_phi(i,1)>=0 phi_n(i,:) = 2*pi - phi_n(i,:) ; end
152 end
153
154 end
```

155 **Constitutive equation function:**

```
156 function [sigmai_r_new,sigmam_new,Chom] = constitutive_latex(n_i,
157   sigmai_r,sigmam,di,dm,Ci_rot,Cm,wi,wm,deltat)
158
159 for  i=1:n_i
160
161 %Objective stress rate
162 Ostressi_r{i}=Ci_rot{i}*di{i};
163 Ostressm=Cm*dm;
164
165 [Ostressi_rC{i}] = cancel_voigt(Ostressi_r{i}); % matrix 3*3
166 [OstressmC] = cancel_voigt(Ostressm); % matrix 3*3
167
168 [wic{i}] = cancel_voigt_antisym(wi{i}); % matrix 3*3
169 [wmc] = cancel_voigt_antisym(wm); % matrix 3*3
170
171 [sigmai_rC{i}] = cancel_voigt(sigmai_r{i}); % matrix 3*3
172 [sigmam_c] = cancel_voigt(sigmam);% matrix 3*3
173
174 sigmai_rc_newC{i}=deltat*(Ostressi_rC{i}- sigmai_rC{i}*wic{i}+ wic{i
175   }*sigmai_rC{i})+sigmai_rC{i}; % t+1
176 sigmam_new =deltat.*(OstressmC - sigmam_c*wmc+ wmc*sigmam_c)+sigmam_c
177   ; % t+1
178
```

```
[sigmai_r_new{i}] = voigt(sigmai_rc_newC{i}); % column
[sigmam_new] = voigt(sigmam_new); %column

end

end
```

Upscaling function:

```
function [Chom_new] = upscaling_latex(n_i,sigma_i,Ci_rot,Cm,A_fib,fi,
    R_fib)
sum_fi_Ci_multi_A_esh= zeros(6,6);

for i=1:n_i
    Ri = expand_antisym(R_fib{i});
    sig = cancel_voigt(sigma_i{i});
for a=1:3
    for j=1:3
        for l=1:3
            for m=1:3
                toto(a,j,l,m) = 0;
                for k=1:3
                toto(a,j,l,m) = toto(a,j,l,m) - sig(a,k) * Ri(k,j,l,m
                    ) + Ri(a,k,l,m) * sig(k,j);
                end
            end
        end
    end
end

% homogenized stiffness tensor
sum_fi_Ci_multi_A_esh=sum_fi_Ci_multi_A_esh+fi(i)*(Ci_rot{i}*A_fib{i}
    + compress(toto)) ;

end

Chom_new = (1-sum(fi))*Cm + sum_fi_Ci_multi_A_esh ;

end
```

Other functions: Functions voigt and cancel-voigt allow to write second-order tensors either as vectors or as matrices, while the compress and expand functions act similarly for fourth-order tensors. The Eshelby-multi function allows to compute the different fourth-order tensors (whose expressions are all analytical).
